# Homologues of xenobiotic metabolizing *N*-acetyltransferases in plant-associated fungi: Novel functions for an old enzyme family

**DOI:** 10.1038/srep12900

**Published:** 2015-08-06

**Authors:** Eleni P. Karagianni, Evanthia Kontomina, Britton Davis, Barbara Kotseli, Theodora Tsirka, Vasiliki Garefalaki, Edith Sim, Anthony E. Glenn, Sotiria Boukouvala

**Affiliations:** 1 Democritus University of Thrace, Department of Molecular Biology and Genetics, Alexandroupolis 68100, Greece; 2 United States Department of Agriculture, Agricultural Research Service, Toxicology & Mycotoxin Research Unit, Richard B. Russell Research Center, 950 College Station Road, Athens, Georgia 30605, USA; 3 University of Oxford, Department of Pharmacology, Mansfield Road, Oxford OX1 3QT, UK

## Abstract

Plant-pathogenic fungi and their hosts engage in chemical warfare, attacking each other with toxic products of secondary metabolism and defending themselves via an arsenal of xenobiotic metabolizing enzymes. One such enzyme is homologous to arylamine *N-*acetyltransferase (NAT) and has been identified in *Fusarium* infecting cereal plants as responsible for detoxification of host defence compound 2-benzoxazolinone. Here we investigate functional diversification of NAT enzymes in crop-compromising species of *Fusarium* and *Aspergillus*, identifying three groups of homologues: Isoenzymes of the first group are found in all species and catalyse reactions with acetyl-CoA or propionyl-CoA. The second group is restricted to the plant pathogens and is active with malonyl-CoA in *Fusarium* species infecting cereals. The third group generates minimal activity with acyl-CoA compounds that bind non-selectively to the proteins. We propose that fungal NAT isoenzymes may have evolved to perform diverse functions, potentially relevant to pathogen fitness, acetyl-CoA/propionyl-CoA intracellular balance and secondary metabolism.

Plant-pathogenic fungi have developed diverse mechanisms to penetrate and colonize hosts, overcoming their physical barriers, chemical defences and complex cellular signalling responses^1^. Plants attack fungi with toxic products of secondary metabolism that can be broadly classified as phytoalexins or phytoanticipins^2^. Invading fungi, in turn, are often capable of overcoming the antimicrobial effects of such chemicals, by employing their xenobiotic metabolizing enzymes, including cytochrome P450, glucosyltransferases and others^3^,^4^.

Two such enzyme activities, essential for the detoxification of benzoxazinoids (antimicrobials produced by maize, wheat and rye), have been mapped at genetic loci *FDB1* and *FDB2* of the endophytic filamentous ascomycete *Fusarium verticillioides*^5^. Benzoxazinoids, like 2,4-dihydroxy-7-methoxy-2H-1,4-benzoxazin-3-one (DIMBOA) and 2,4-dihydroxy-2H-1,4-benzoxazin-3-one (DIBOA), are phytoanticipins generated via a well-characterized biosynthetic pathway^6^,^7^, released as aglycones that rapidly degrade into the corresponding benzoxazolinones 6-methoxy-2-benzoxazolinone (MBOA) and 2-benzoxazolinone (BOA). These, too, exert toxic effects to unwelcome microorganisms, herbivores or weeds by reacting with nucleophilic groups (e.g. -NH_2_ and -SH) of biomolecules^7^. Endophytic fungi associated with cereal plants, like the maize pathogen *F. verticillioides*, have adapted their xenobiotic metabolism to effectively detoxify MBOA and BOA, by employing the metabolic activities encoded at the *FDB1* and *FDB2* loci^5^,^8^. The former activity mediates BOA hydrolysis to the intermediate compound 2-aminophenol (2AP), while the latter involves a *N*-malonyltransferase catalysing the conversion of 2AP to the non-toxic compound *N-*(2-hydroxyphenyl)malonamic acid (HPMA)^5^. Cloning of the *N-*malonyltransferase gene revealed a sequence highly homologous to arylamine *N*-acetyltransferase (NAT), while deletion of the *FDB2* locus further demonstrated a secondary branch to the metabolic pathway, potentially involving another NAT homologue catalysing *N-*acetylation of 2AP to *N-*(2-hydroxyphenyl)acetamide (HPAA)^9^. Genomic analysis identified a total of four putative *NAT* loci in *F. verticillioides*, while screening of an additional 145 fungal genomes annotated multiple putative homologues in various ascomycetes within the subphylum of Pezizomycotina^10^,^11^.

NAT was originally identified by Nobel Laureate Fritz Lipmann and his co-workers^12^, as the hepatic enzymatic activity responsible for the acetyl-coenzyme A (CoA) dependent *N-*acetylation of arylamines (EC 2.3.1.5). It was subsequently demonstrated that the same enzyme entity also bears *N-*hydroxyarylamine *O-*acetyltransferase (EC 2.3.1.118) and arylhydroxamic acid *N*,*O*-acetyltransferase (EC 2.3.1.56) activities^13^. Pharmacological work demonstrated that human NAT is also active against aromatic hydrazines, such as the anti-tubercular drug isoniazid (INH), and polymorphic acetylation of INH and other common drugs has been the subject of intense pharmacogenetic research^14^. Polymorphic NAT activity has additionally been associated with xenobiotic-induced carcinogenicity of the bladder^15^. The mechanism of NAT-mediated *N-*acetylation has been studied in humans and laboratory mammals, as well as in prokaryotes, including mycobacteria^16^. Irrespectively of their kingdom of origin, all characterized NAT homologues appear to perform their enzyme function via a conserved “catalytic triad” (typically formed by residues cysteine-histidine-aspartate) similar to that of cysteine proteases^17^,^18^. Genomic surveys indicate NAT homologues in phylogenetically diverse organisms, although gaps in the evolution of the *NAT* gene family appear to exist, most notably in plants^10^,^19^. NATs are well-represented in fungi, particularly plant-associated ascomycetes which are predicted to harbour more than one putative paralogue in their genome^10^,^11^. Recent studies have also explored *N-*acetylation by fungal NATs as a potential route for detoxification of environmental pollutants^20^,^21^.

Our previous work on the BOA-detoxification pathway of *F. verticillioides*^9^, points towards possible functional diversification of NAT enzymes in plant-pathogenic fungi. Here, we characterize the NAT homologues of five representative species of *Fusarium* and *Aspergillus*, four of which (including *F. verticillioides*) compromise various crops. Our results support that different NAT isoenzymes of plant-associated ascomycetes are not just the product of genetic redundancy, but may have evolved to perform distinct functions.

## Results

### Characterization of *NAT* loci in plant-associated ascomycetes

Our previous survey^10^ predicted 16 putative *NAT* loci in the sequenced genomes of the maize pathogen *Fusarium verticillioides* (teleomorph *Gibberella moniliformis*; strain FGSC 7600; 4 *NAT* loci), the wheat pathogen *Fusarium graminearum* (teleomorph *Gibberella zeae*; strain PH-1; 3 *NAT* loci), the tomato pathogen *Fusarium oxysporum* f.sp. *lycopersici* (strain FOL 4287; 4 *NAT* loci), the grain contaminant *Aspergillus flavus* (strain NRRL 3357; 4 *NAT* loci) and the laboratory model fungus *Aspergillus nidulans* (teleomorph *Emericella nidulans*; strain FGSC A4; 1 *NAT* locus). To validate the computational annotations, we undertook PCR amplification and sequencing of those loci from genomic DNA and cDNA isolated from the five ascomycetes, followed by computational alignment of the identified sequences to determine the exon-intron structure of each *NAT* gene. Thirteen *NAT* loci were confirmed to generate transcripts with open reading frames (ORFs) that translate into peptide sequences with the characteristic conserved elements of NAT enzymes^10^,^17^,^18^ (Table 1, Supplementary Fig. S1). In contrast, amplification from cDNA with primers specific for the predicted *NAT4* loci of *F. verticillioides* and *A. flavus* identified hypothetical ORFs compromised by multiple nonsense mutations, suggesting that those two loci may be transcribing pseudogenes. Moreover, the *NAT1* locus of *A. flavus* is most likely not transcribed, under the standard culture conditions applied, since different combinations of primers generated specific amplification products from genomic DNA, but not from cDNA. The ORFs of fungal *NAT* genes, shown to generate protein-coding transcripts, were cloned and expressed in *Escherichia coli*, providing recombinant proteins with the expected size upon SDS-PAGE (Supplementary Fig. S2).

### Acyl-coenzyme A selectivity of fungal NAT enzymes

**Figure 1:**
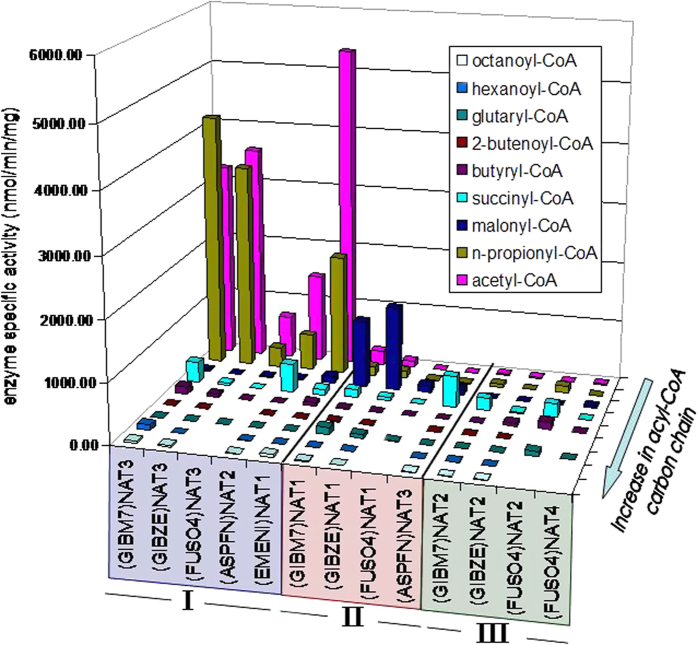
Activity of fungal NAT enzymes with different acyl-coenzyme A compounds. Overview of the acyl-CoA selectivity pattern observed for the recombinant NAT isoenzymes of *F. verticillioides* (*G. moniliformis*-GIBM7), *F. graminearum* (*G. zeae*-GIBZE), *F. oxysporum* f.sp. *lycopersici* (FUSO4), *A. flavus* (ASPFN) and *A. nidulans* (*E. nidulans*-EMENI). Functional homologues are grouped together within coloured boxes labelled I-III. Each enzyme was assayed against a series of acyl-CoA compounds, used as acyl-group donors in reactions with 5-aminosalicylate as acceptor substrate. The results for each set of assays are presented in Supplementary Fig. S3.

The *N-*malonyltransferase encoded by the *FDB2* locus in *F. verticillioides* has been assigned symbol NAT1^9^,^10^. Here, we show that this isoenzyme selectively employs malonyl-CoA to catalyze *N-*malonyl transfer to an arylamine, and that this enzymatic reaction is specific to the NAT1 homologues of *F. verticillioides* [(GIBM7)NAT1] and *F. graminearum* [(GIBZE)NAT1], the two fungi in our panel that are naturally exposed to BOA through their endophytic association with cereals. Homologous NAT isoenzymes are also predicted for the tomato pathogen *F. oxysporum* f.sp. *lycopersici* [(FUSO4)NAT1] and potentially for the non-endophytic maize pathogen *A. flavus* [(ASPFN)NAT3], although the *N-*malonyltransferase activity detected with those recombinant proteins was very low. No *N-*malonyltransferase homologue was found in *A. nidulans*, a fungus not associated with plants (Fig. 1 and Supplementary Fig. S3).

The 13 fungal NAT proteins, expressed here in recombinant form, were subjected to enzymatic activity assays with a series of acyl-CoA compounds ranging in acyl-chain length from acetyl- to octanoyl-CoA. The screen identified three distinct groups (I-III) of NAT homologues in the five ascomycetes investigated (Fig. 1 and Supplementary Fig. S3). Each fungus has one NAT isoenzyme (group I) with typical *N-*acetyltransferase activity, and those homologues are also highly selective for n-propionyl-CoA. Group II comprises the *N-*malonyltransferase homologues described above, while the NAT proteins in group III show no specific preference for any of the acyl-CoAs tested. Malonyl-CoA was employed exclusively by the *N-*malonyltransferases, which also provided minimal activity with acetyl- and n-propionyl-CoA. Low levels of activity were also detected with succinyl-CoA for several NAT isoenzymes, but no specific selectivity pattern was evident among homologues. Other acyl-CoA compounds provided residual or no activity with fungal NATs (Fig. 1 and Supplementary Fig. S3).

**Figure 2:**
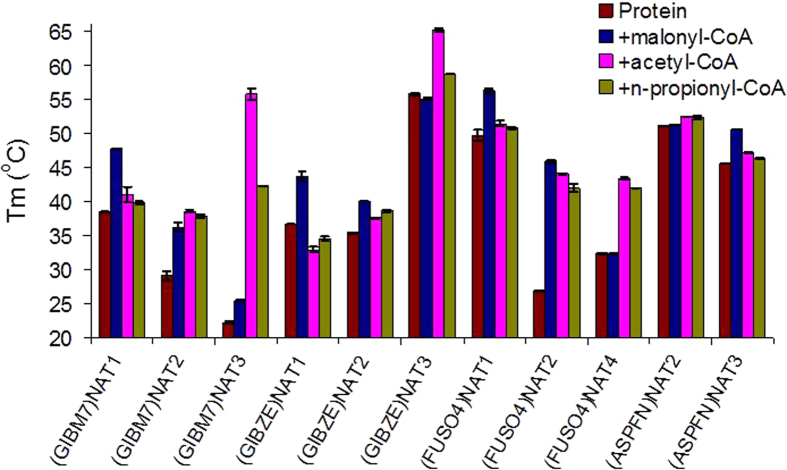
Effect of acyl-coenzyme A compounds on the Tm of fungal NAT proteins. Overview of Tm values determined by differential scanning fluorimetry for recombinant NAT isoenzymes of *F. verticillioides* (*G. moniliformis*-GIBM7), *F. graminearum* (*G. zeae*-GIBZE), *F. oxysporum* f.sp. *lycopersici* (FUSO4) and *A. flavus* (ASPFN), in the absence or presence of various acyl-CoAs. Two replicate experiments were performed, generating overlapping curves for which the average Tm (± standard deviation) is shown. The results for each set of experiments are presented in Supplementary Fig. S4.

Binding of acyl-CoAs to fungal NATs was investigated using differential scanning fluorimetry (DSF), a technique that detects changes in the thermal stability of recombinant proteins upon interaction with their specific ligands^22^. The method was applicable with all recombinant NAT proteins in our panel, except the NAT3 of *F. oxysporum* [(FUSO4)NAT3] and the NAT1 of *A. nidulans* [(EMENI)NAT1] which were recovered at less optimal levels of yield and purity (Supplementary Fig. S2). The Tm values of different fungal NAT homologues varied considerably from around 20 to over 55 ^°^C, with the NAT3 homologues of *F. verticillioides* [(GIBM7)NAT3] and *F. graminearum* [(GIBZE)NAT3] at opposite extremes (Fig. 2 and Supplementary Fig. S4). The addition of acyl-CoAs increased the Tm values of NAT proteins, with a pattern that reflected exactly the results of our activity assays for group I *N-*acetyl/*N-*propionyltransferases and group II *N-*malonyltransferases. In the case of group I homologues [(GIBM7)NAT3, (GIBZE)NAT3 and (ASPFN)NAT2], the shift in Tm was always greater with acetyl-CoA, intermediate with n-propionyl-CoA and minimal with malonyl-CoA. Conversely, for group II *N-*malonyltransferases [(GIBM7)NAT1, (GIBZE)NAT1, (FUSO4)NAT1 and (ASPFN)NAT3], the increase in Tm was substantial only with malonyl-CoA (Fig. 2 and Supplementary Fig. S4).

The results of the DSF analysis indicated that the NAT homologues of group III, appearing as functionally redundant in our activity assays with various acyl-CoAs (Fig. 1), are in fact capable of binding acetyl-, n-propionyl-, malonyl- and succinyl-CoA [(GIBM7)NAT2, (GIBZE)NAT2 and (FUSO4)NAT2]. The only deviation within group III was observed for the NAT4 isoenzyme of *F. oxysporum* [(FUSO4)NAT4], where the shift to Tm was evident with acetyl- and n-propionyl-CoA and absent with malonyl-CoA (Fig. 2 and Supplementary Fig. S4). It is possible that this particular isoenzyme of *F. oxysporum* is distinct from other fungal NAT homologues in our experimental panel.

### Substrate selectivity of fungal NAT enzymes

**Figure 3:**
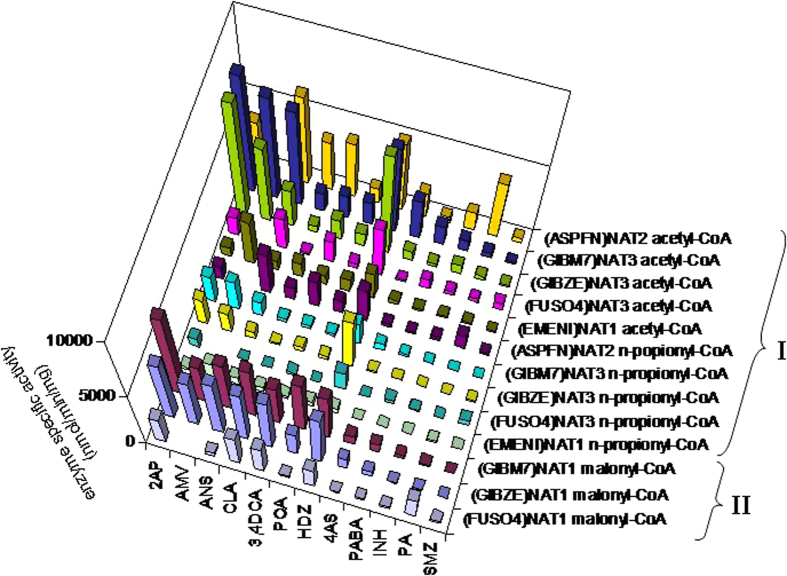
Activity of fungal NAT enzymes with different acceptor substrates. Overview of the acceptor substrate selectivity pattern observed for group I and II isoenzymes of *F. verticillioides* (*G. moniliformis*-GIBM7), *F. graminearum* (*G. zeae*-GIBZE), *F. oxysporum* f.sp. *lycopersici* (FUSO4), *A. flavus* (ASPFN) and *A. nidulans* (*E. nidulans*-EMENI). Recombinant NAT proteins were assayed with selective acyl-CoAs against a panel of arylamine and arylhydrazine substrates, and the results for each set of assays are presented in Supplementary Fig. S5. The full chemical names of compounds are: 2-aminophenol (2AP), 4-aminoveratrole (AMV), 4-anisidine (ANS), 4-chloroaniline (CLA), 3,4-dichloroaniline (3,4DCA), 4-phenoxyaniline (POA), hydralazine (HDZ), 4-aminosalicylate (4AS), 4-aminobenzoate (PABA), isoniazid (INH), procainamide (PA) and sulphamethazine (SMZ).

To assess the enzymatic activity of recombinant fungal NAT proteins towards different substrates, assays were performed with the preferred acyl-CoA of each isoenzyme (as identified by the enzymatic and DSF analyses of the previous section) against a representative panel of arylamine and arylhydrazine compounds^23^. Group I homologues provided higher enzyme activities with acetyl-CoA and lower with n-propionyl-CoA, but the overall specificity pattern with respect to acceptor substrate was similar with both acyl-group donor compounds (Fig. 3 and Supplementary Fig. S5).

Our screening showed that 2AP, the substrate of NAT1-mediated *N-*malonyltransfer leading to BOA detoxification in *F. verticillioides*^9^, generates high levels of activity with several group I and group II homologues. Toxic substituted anilines, such as the haloanilines 4-chloroaniline (CLA) and 3,4-dichloroaniline (3,4DCA), and the alkoxyanilines 4-anisidine (ANS) and 4-aminoveratrole (AMV), were also effective substrates of fungal NAT enzymes. On the other hand, 4-aminosalicylate (4AS), 4-aminobenzoate (PABA), procainamide (PA), sulphamethazine (SMZ) and INH, all well-characterized pharmaceutical compounds metabolized by mammalian NAT isoenzymes, were demonstrated to be poorer substrates of fungal NATs. Of the two arylhydrazines used, the antihypertensive hydralazine (HDZ) generated considerably higher levels of NAT enzymatic activity than the anti-tubercular INH (Fig. 3 and Supplementary Fig. S5).

### Endogenous NAT activities of fungi and effects of xenobiotics

**Figure 4:**
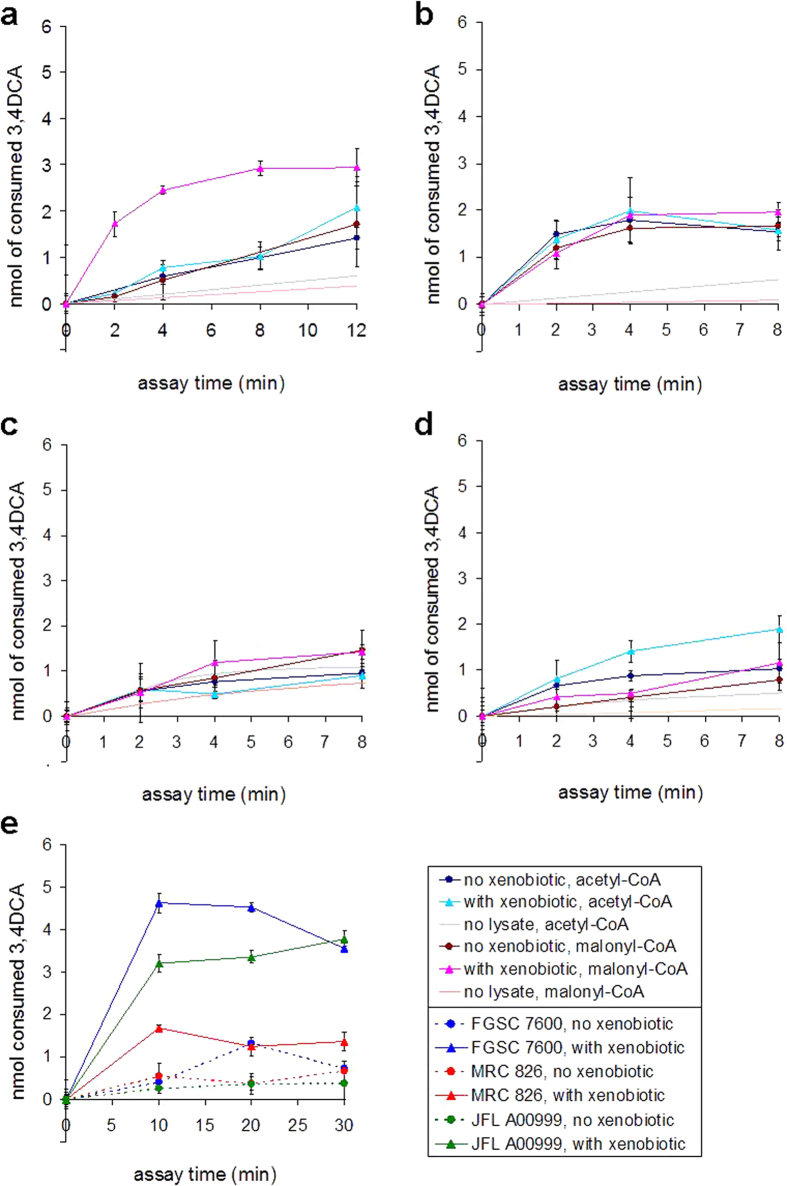
NAT enzymatic activities in fungal cell extracts upon xenobiotic exposure. Erlich’s reagent was used to measure NAT activity in fungal soluble extracts, following enzyme assays with 3,4-dichloroaniline (3,4DCA) and either acetyl- or malonyl-CoA. The graphs show comparison of NAT enzymatic activities measured in cell extracts from cultures challenged for 2 h with xenobiotics (mixture of 2-benzoxazolinone and 3,4DCA, each at 25 μg/ml), relative to extracts prepared from cultures grown in standard medium. Control assays, without cell extract, are also shown. Each data point is the average value of three replicates ± standard deviation. Results are shown for assays performed with cell extracts from *F. verticillioides* (*G. moniliformis*) strain FGSC 7600 **(a)**, *F. graminearum* (*G. zeae*) strain PH-1 **(b)**, *F. oxysporum* f.sp. *lycopersici* strain FOL 4287 **(c)** and *A. flavus* strain NRRL 3357 **(d)**. The effects of xenobiotics on NAT enzymatic activity measured with malonyl-CoA in cell extracts from *F. verticillioides* strains FGSC 7600, MRC 826 and JFL A00999 are also compared **(e)**.

Cellular *N-*acetyltransferase and *N-*malonyltransferase activities were measured with 3,4DCA in soluble extracts of *F. verticillioides*, *F. graminearum*, *F. oxysporum* f.sp. *lycopersici* and *A. flavus*, grown in liquid media with or without xenobiotics. The fungi were challenged with a mixture of BOA and 3,4DCA, in order to achieve induction of the BOA detoxification pathway^9^ and potentially other cellular NAT enzymes. In *A. nidulans*, the yield of total soluble protein was reduced by over 2.5-fold (4.4 *vs.* 11.7 mg) in extracts from cultures with *vs.* without xenobiotics, suggesting that the fungus is unable to tolerate exposure. Xenobiotic challenge had only minimal effect (up to 1.5-fold increase) on the *N*-acetyltransferase activity of the four other fungi (Fig. 4).

In *F. verticillioides* strain FGSC 7600, the *N-*malonyltransferase activity was relatively low (1 nmol/min/mg), but increased sharply (by 8-fold) upon challenge with xenobiotics (Fig. 4a). Induction was also evident with *F. verticillioides* strains MRC 826 and JFL A00999 (Fig. 4e). In *F. graminearum*, *N-*malonyltransferase activity was higher (5.3 nmol/min/mg), but xenobiotic exposure had no apparent effect. *N-*malonyltransferase activity was also present in *F. oxysporum* (2.3 nmol/min/mg) and was not affected by the xenobiotics. In *A. flavus*, the *N-*malonyltransferase activity was low (0.8 nmol/min/mg), but xenobiotics caused a moderate (2.5-fold) increase (Fig. 4).

**Figure 5:**
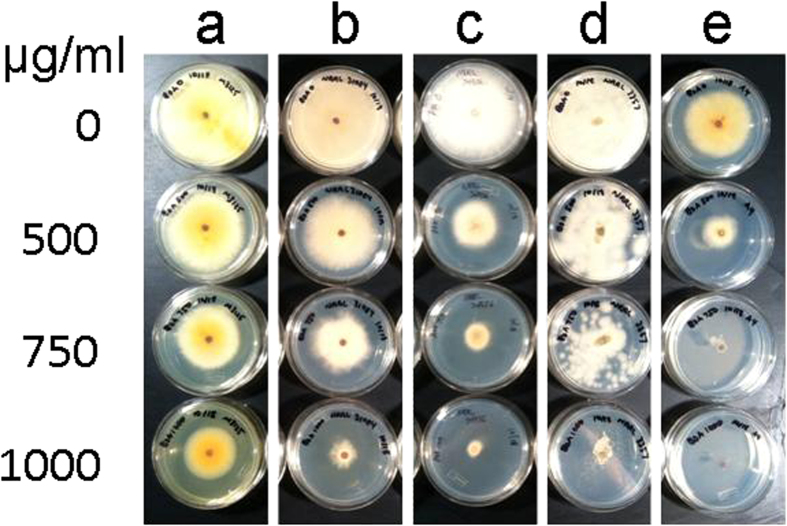
Fungal tolerance of 2-benzoxazolinone. *F. verticillioides* (*G. moniliformis*) strain FGSC 7600 **(a)**, *F. graminearum* (*G. zeae*) strain PH-1 **(b)**, *F. oxysporum* f.sp. *lycopersici* strain FOL 4287 **(c),** *A. flavus* strain NRRL 3357 **(d)** and *A. nidulans* (*E. nidulans*) strain FGSC A4 **(e)** were grown on standard agar medium supplemented with up to 1000 μg/ml of 2-benzoxazolinone. Cultures are shown after 5 days of incubation.

The results of the enzymatic assays measuring endogenous *N-*malonyltransferase activity are reflected in the ability of the ascomycetes to tolerate BOA in solid culture media. *F. verticillioides* grew effectively on media with up to 1 mg/ml BOA, a toxic concentration for other fungi. *F. graminearum* also tolerated BOA, but radial growth was slower and toxicity was evident at 1 mg/ml concentration of the compound. *F. oxysporum* and *A. flavus*, on the other hand, were sensitive to BOA, with delayed growth evident on media with lower concentrations of the compound. Consistent with our observations above, growth of *N-*malonyltransferase deficient *A. nidulans* was much reduced at the lowest concentration of BOA, and the higher concentrations completely inhibited growth (Fig. 5).

## Discussion

We have investigated 16 *NAT* loci from five filamentous ascomycetes, including four plant pathogens and three mycotoxigenic fungi of agricultural importance. Three of those loci are apparently non-functional, while the remaining 13 loci were demonstrated to comprise ORFs generating recombinant proteins with size and deduced amino acid sequences characteristic of prokaryotic and eukaryotic NAT homologues. Earlier studies of eukaryotic *NAT* genes have been confined mainly to humans and model mammals, where *NAT* ORFs are always contained in a single exon, although upstream non-coding exons have been experimentally characterized and alternative splicing in the 5′-untranslated region has been observed^24^,^25^. Segmented NAT ORFs, demonstrated experimentally here, have been computationally predicted previously for certain fungi, protists and lower chordates^10^,^11^.

Specificity of our 13 fungal NAT proteins was elucidated by enzymatic activity assays with nine different acyl-CoAs as donor substrates, followed by screens against a panel of arylamine and arylhydrazine acceptor substrates. To our knowledge, this dataset represents the largest assemblage of comparative specific activity analyses ever presented for NAT enzymes, revealing unique specificity profiles and supported by DSF experiments. We propose that fungal NATs may be divided into at least three biochemically distinct groups (I-III) of homologues, depending on their acyl-CoA specificity and postulated function. Such classification is exactly reflected in our previous phylogenetic analysis^10^, which indicated distinct, well-supported lineages of group I, II and III homologues. The group I homologues of *A. flavus* and *A. nidulans* formed a strongly supported lineage with other *Aspergillus* orthologues that is paraphyletic to the group I homologues of the three *Fusarium* species. Particularly interesting was the lineage of group II homologues, which appeared to include maize and wheat pathogens additional to *Fusarium*. Consistent with the biochemical analysis, the protein sequence phylogeny suggested that *F. oxysporum* NAT4 has diverged from the other *Fusarium* group III homologues. Lastly, although *A. flavus* NAT3 demonstrated biochemical characteristics of group II homologues, it appeared phylogenetically more related to group III. Continued investigation into these deviating homologues may enable a more accurate classification of their particular functions.

We demonstrate that the group II homologues of *F. verticillioides* and *F. graminearum* are *N*-malonyltransferases selectively utilizing malonyl-CoA to acylate arylamines, including the 2AP intermediate formed during hydrolysis and detoxification of BOA. To our knowledge, these are the first NAT isoenzymes conclusively shown to have specificity for malonyl-CoA instead of the typical acetyl-CoA. *F. oxysporum* and *A. flavus* also appear to possess a NAT homologue preferentially binding malonyl-CoA, but *N-*malonyltransferase activity was low with arylamine in preparations of both recombinant proteins and cell lysates. Upon xenobiotic exposure, *F. verticillioides* demonstrated substantial induction of arylamine *N-*malonyltransferase activity, reflecting the ability of this fungus to readily overcome BOA concentrations which are toxic to other fungi. *F. graminearum*, on the other hand, appeared to rely on its substantial, albeit non-inducible, arylamine *N-*malonyltransferase activity to manage delayed growth on BOA. In contrast, *F. oxysporum* and *A. flavus* were both more sensitive to BOA, presumably due to their low endogenous arylamine *N-*malonyltransferase activity, although other possible dysfunctions in their postulated BOA detoxification pathway cannot be ruled out. *A. nidulans* lacks a group II NAT homologue and was more sensitive to BOA than the other fungi. Experiments designed to accurately quantify the effects of xenobiotics on *NAT* gene transcriptional expression are currently underway, expanding the present biochemical analysis.

From this and our previous studies^9^, it is evident that group II NAT homologues have diverged to serve the purpose of *Fusarium* endophytic survival in the BOA-enriched environment of cereal plant tissues. Other ascomycetes, with less specific host associations, exhibited lower *N-*malonyltransferase activity (as in the case of non-endophytic pathogens *F. oxysporum* and *A. flavus*) or completely lacked the corresponding *NAT* gene (as in the case of *A. nidulans*). Interference with the BOA detoxification pathway, either via deletion of the *NAT1* gene^9^,^26^ or co-culture with antagonistic *Bacillus* bacteria^27^, has been shown to compromise the BOA tolerance of endophytic *Fusaria* infecting cereal plants. Furthermore, a recent study^26^ demonstrates reduction in virulence of the wheat pathogen *F. pseudograminearum*, upon deletion of its *NAT1* orthologue. NAT1 inhibition might thus represent a novel strategy to control maize and wheat pathogens, and available panels of NAT inhibitors^16^ could be relevant.

All five fungi studied were shown to possess one group I homologue with preference for acetyl-CoA, as expected by an archetypal NAT enzyme. Consistent with previous observations for *Podospora anserina*^20^, these homologues exhibited relatively low activity with established pharmaceutical substrates of NAT enzymes, but were highly active towards toxic substituted anilines, including 3,4DCA, a derivative compound of commonly applied agrochemicals and the subject of several bioremediation studies^28^,^29^. In cell lysates of our fungi, particularly *F. graminearum*, *N*-acetyltransferase activity was readily detectable with arylamine, but was apparently non-inducible upon challenge with BOA and 3,4DCA. Collectively, these data imply a role for group I *N-*acetyltransferases distinct from group II *N-*malonyltransferases. Moreover, the apparent constitutive expression of arylamine *N-*acetyltransferase activity in both challenged and unchallenged cultures suggests that group I NAT homologues may facilitate endogenous metabolism in a manner independent of xenobiotic acylation.

There are various hypotheses as to the possible endogenous roles of NAT enzymes. In human and transgenic mice, certain NAT homologues have been investigated for possible involvement in folate metabolism^30^,^31^. In actinobacteria, some *NAT* genes have been localized within operons regulating cholesterol catabolism to propionyl-CoA^32^,^33^, an acyl-group donor substrate of NATs, as shown by this and previous studies^23^,^34^. In tuberculous mycobacteria, the genes co-localizing with *NAT* are essential for survival of the pathogen on cholesterol and in host macrophage^35^, while *NAT* gene deletion in *Mycobacterium bovis* BCG compromises mycolic acid biosynthesis and cell wall integrity^36^. Here, we show that propionyl-CoA is employed as acyl-group donor substrate exclusively by group I NAT homologues of all five fungi examined. In contrast, group II and III homologues were essentially inactive towards propionyl-CoA, although group III homologues appeared to non-selectively bind the compound.

Propionyl-CoA is generated in microbial cells through oxidation of odd-chain fatty acids or catabolism of valine, isoleucine, and methionine. Uptake of propionate from environmental sources can also produce propionyl-CoA via thioesterification. Excess accumulation of propionyl-CoA is toxic to cells, due to inhibition of several primary metabolic enzymes, and propionate is commonly used as a food preservative to suppress microbial growth^37^. Additionally, propionyl-CoA accumulation in fungi has been shown to inhibit the biosynthesis of polyketide-derived secondary metabolites, such as ochratoxin, sterigmatocystin, and spore pigment^37^,^38^,^39^,^40^. Inhibition of polyketide formation is likely due to an intracellular imbalance of the ratio of propionyl-CoA to acetyl-CoA. For degradation of propionyl-CoA, fungi utilize the methylcitrate cycle, in which methylcitrate synthase condenses propionyl-CoA with oxaloacetate to generate methylcitrate and, ultimately, compounds used in primary metabolic pathways^41^. As speculated^42^, plant pathogenic fungi likely encounter carbon sources during plant infection that result in propionyl-CoA generation. Metabolic profiling of soybean roots inoculated with *Fusarium tucumaniae* showed pathogen-induced accumulation of valine and other amino acids, at early time points in the infection^43^. These data are consistent with the quantification of dipeptides (composed of branched-chain amino acids, predominantly leucine and isoleucine) in *Arabidopsis* roots, where they were abundant with significantly higher amounts in the epidermis and endodermis compared with other root cell types^44^. Degradation of these dipeptides would provide an abundance of isoleucine and valine amino acids, which could result in surplus accumulation of propionyl-CoA in a fungal pathogen infecting the roots. Given their dual preference for acetyl-CoA and propionyl-CoA, group I NAT homologues could function to alleviate toxicity when propionyl-CoA is in excess. This hypothesis could be tested using the methylcitrate synthase deficient *A. nidulans* mutant developed by Brock and colleagues^41^. This strain suffers from elevated intracellular levels of propionyl-CoA, resulting in toxicity and reduced mycotoxin production^38^,^39^. Loss of group I NAT homologues might have similar effects. We are currently pursuing development of genetically engineered strains of *F. verticillioides*, to investigate the effects of *NAT* gene inactivation or overexpression on cell viability, physiology and secondary metabolism. We speculate that group I NAT enzymes may act to maintain a balanced propionyl-CoA to acetyl-CoA ratio within cells.

Our screen of recombinant NAT proteins suggested a third group of homologues in plant-associated fungi, with essentially no activity towards any of the acyl-CoAs tested. However, these group III homologues were readily expressed and purified in substantial yields as soluble proteins in *E. coli*. Moreover, DSF analysis demonstrated their ability to non-selectively bind acetyl-, malonyl-, propionyl- and succinyl-CoA, the only deviation being the NAT4 of *F. oxysporum* which exhibited group I-like acyl-CoA binding specificity but no enzyme activity. Transcription of the corresponding genes was also verified by PCR from fungal cDNA. We propose that group III NAT homologues may have an as yet uncharacterized function.

Functional divergence of NATs has been reported before in the filamentous actinomycete *Amycolatopsis mediterranei*, where one NAT homologue (encoded by *RifF* locus of the rifamycin biosynthetic gene cluster) performs the final cyclization step of rifamycin and its consequent release from the polyketide synthase (PKS). This is an unexpected reaction for NAT, both in terms of excessive size of accommodated substrate and apparent acyl-CoA independence of the enzyme^45^. Prokaryotic filamentous actinomycetes demonstrate immense potential for secondary metabolism^46^ and our database searches suggest that many of their sequenced representatives (particularly *Streptomycetes*) harbour multiple *NAT* genes in their genome. This is very similar to the pattern observed for eukaryotic filamentous ascomycetes, where *NAT* genes are well-represented in sequenced genomes of Pezizomycotina (particularly endophytic and soil fungi with diverse metabolic capabilities), but absent in Saccharomycotina yeasts^10^,^11^. A bioinformatics analysis comparing Pezizomycotina *vs.* Saccharomycotina genomes indicates that genes present in the former and absent in the latter group of ascomycetes are primarily relevant to plant biomass decomposition, secondary metabolism and amino acid (particularly valine, leucine and isoleucine) degradation^47^. This potentially implicates Pezizomycotina NATs in xenobiotic detoxification, acetyl-CoA/propionyl-CoA homeostasis and/or secondary metabolism.

There is no solid evidence whether *NAT* genes might be part of polyketide biosynthetic gene clusters in the five ascomycetes examined. One recent study has placed *NAT3* of *A. flavus* in a putative biosynthetic gene cluster predicted to be responsible for production of a polyketide that is some type of pigment^48^. The cluster has been experimentally studied in *A. oryzae*, but the role of *NAT3* (*aoiL* locus in the study) is not clear^49^. The most similar orthologue of the PKS gene defining the postulated *NAT3*-containing cluster in *Aspergillus* is found on the gene cluster responsible for production of the red pigment bikaverin in *Fusarium*^50^. The bikaverin cluster does not bear any *NAT* orthologue; however, recent bioinformatics analyses suggest that certain *NAT* genes in *Fusarium* are contained within subtelomeric chromosomal regions which are known to be highly variable and harbour genes involved in secondary metabolism and adaptation of fungi to environmental stimuli^51^. Biosynthesis of polyketide-type secondary metabolites proceeds via iterative condensation and chain elongation, based on carbon units typically delivered by acetyl-CoA and malonyl-CoA^52^, again suggesting a possible role of NATs as mediators of intracellular acyl-CoA homeostasis.

This investigation provides insight into the distribution and function of NAT enzymes in mycotoxigenic fungi of global concern for agriculture and the food industry^53^. Recent comparative genomic analyses indicate that the full functional diversity and potential of many microbial proteins has not yet been demonstrated, as laboratory culture conditions provide limited opportunity for discovery of complex biochemical pathways related to secondary metabolism and cell adaptation to variable environmental stresses or sources of nutrients^54^. We believe such investigations may be relevant to fungal NAT enzymes and propose research directions stemming from comparative investigations of different groups of homologues.

## Methods

### Fungal strains

Fungi used in the study are described below, along with the strains examined (synonymous strain identifiers in parentheses), host organisms and geographical location of origin^8^. *Fusarium verticillioides* (teleomorph *Gibberella moniliformis*), sequenced strain FGSC 7600 (JFL A00149/FRC M3125/NRRL 20956), maize, California, USA^55^; strain JFL A00999 (FGSC 7603/ATCC 201261/FRC M3703/NRRL 20984), maize, Indiana, USA; strain MRC 826 (FRC M1325/NRRL 13447), maize, South Africa. *Fusarium graminearum* (teleomorph *Gibberella zeae*), sequenced strain PH-1 (NRRL 31084/ATCC MYA-4620/FGSC 9075), wheat, Michigan, USA^56^. *Fusarium oxysporum* f.sp. *lycopersici*, sequenced strain FOL 4287 (NRRL 34936/CBS 123668/FGSC 9935), tomato, Spain^55^. *Aspergillus flavus*, sequenced strain NRRL 3357 (ATCC 200026/FGSC A1120/JCM 12722/SRRC 167), maize and peanut, USA^57^. *Aspergillus nidulans* (teleomorph *Emericella nidulans*), sequenced strain FGSC A4 (ATCC 38163/CBS 112.46/NRRL 194/M139), Glasgow soil sample^58^.

### Culture conditions and preparation of fungal cell extracts

Cultures (50 ml) were initiated from frozen stocks (−80 ^o^C, 15% v/v glycerol), and the fungi were grown in media and at temperatures generally regarded as standard conditions for the respective genera. Starter cultures of *Fusarium* were grown in potato dextrose broth (Fluka) for 3 days at 25 ^o^C in the dark (200 rpm). Starter cultures of *Aspergillus* were grown in complete medium (Fluka) for 24 h at 37 ^o^C in the dark (200 rpm). Twenty flasks (each with 50 ml of appropriate medium), per experiment, were subsequently inoculated with 1 ml (*Fusarium*) or 6–7 ball-like clumps of hyphae (*Aspergillus*) from the starter cultures, and the new cultures were incubated under the same conditions. Half of the flasks then received a mixture of xenobiotics (fresh preparations of BOA and 3,4DCA, each compound added to the liquid culture at 25 μg/ml final concentration) and were incubated for 2 h, with cultures then placed on ice for immediate harvest. Cultures without xenobiotics were treated in exactly the same way for use as controls. Both xenobiotic-amended and control cultures contained 1% v/v ethanol. The multiple flasks of fungal growth for each treatment were combined together, centrifuged (6,000 xg, 10 min, 4 ^o^C) to remove most of the medium, and the fungal pellet was filtered (Whatman 41 ashless filter paper) under vacuum. The recovered cell paste was flash-frozen and ground in liquid nitrogen, using mortar and pestle. Cell extracts were used to isolate DNA, total RNA or total soluble protein, as described in the sections that follow. *Fusarium* was also grown on potato dextrose agar solid medium at 25 ^o^C, and *Aspergillus* on agar minimal medium at 37 ^o^C (both media from Fluka), with or without xenobiotic (0–1 mg/ml BOA). For short-term induction experiments, the time of exposure (2 h) and the concentration of xenobiotics (50 μg/ml total) were empirically determined, so that the endogenous NAT activities could be quantified without toxic effects on fungal cells. In contrast, for agar-based tolerance assays, higher doses (up to 1 mg/ml) of xenobiotic were justified, to the level of toxicity, in order to distinguish tolerant from sensitive fungi.

### Characterization of fungal *NAT* genes

The sequences of putative fungal *NAT* genes were retrieved from genomic databases and computationally annotated as described previously^10^. Isolation of nucleic acids was carried out from cell extracts, using the DNeasy (for DNA) or RNeasy (for RNA) Plant Mini Kit (Qiagen) according to the manufacturer’s instructions. The RNA isolation procedure included on-column treatment with deoxyribonuclease I to eliminate DNA. The preparations were assessed on a Nanodrop ND-1000 spectrophotometer (Thermo Scientific) and by microfluidic analysis of RNA integrity (RNA integrity numbers of 6–8) on an Agilent 2100 Bioanalyzer (Agilent Technologies). Complementary DNA was generated from 1 μg/reaction of RNA, using AMV reverse transcriptase (Roche) and oligo [dT]_16_ primer. Mock reactions without reverse transcriptase were performed to confirm that RNA preparations were devoid of genomic DNA. High-fidelity *Pfu*-DNA polymerase (Finnzymes) and combinations of primers shown in Supplementary Table 1 were used to PCR-amplify fungal *NAT* genes from genomic DNA or cDNA template, followed by direct sequencing (GATC Biotech, Germany) of the products with the same primers. Validated genomic and transcribed *NAT* sequences were aligned with the BioEdit Sequence Alignment Editor 7.0.5.3, in order to determine the exon-intron structure of each gene. All fungal *NAT* sequences were submitted to the European Nucleotide Archive (http://www.ebi.ac.uk/ena). Gene symbols are according to the recommendations of the Arylamine *N*-acetyltransferase Gene Nomenclature Committee^59^, with specific details for naming fungal NATs provided in the Supplementary Methods.

### Cloning of fungal *NAT* genes

Amplified products of the complete ORFs of fungal *NAT* genes (generated from cDNA or, in the case of intronless *NAT* genes, from genomic DNA) were A-tailed at the 3′-ends with *Taq-*DNA polymerase, then ligated to *Eco*RV-digested and T-tailed pGEM-5zf(+) cloning vector. Following transformation into *E. coli* JM109 competent cells, colonies were PCR-screened for insert orientation with vector-specific forward primer (T7-promoter) and the appropriate gene-specific reverse primer (Supplementary Table 1). Clones passing this test were sequenced from both ends with vector-specific primers (T7-promoter and SP6-promoter). For recombinant protein expression, the intronless ORFs of fungal *NAT* genes were recovered from validated pGEM-5zf(+) constructs, by cutting the vector at restriction sites flanking the beginning (*Sac*II) and the end (*Not*I) of each insert. The excised fragments were then ligated to a *Nde*I-*Sac*II adaptor (Supplementary Table 1), providing the *Nde*I-compatible end required for in-frame incorporation of the *Sac*II/*Not*I-digested inserts into *Nde*I/*Not*I-digested pET28b(+) recombinant expression vector (Novagen). The adaptor allowed expression of NAT proteins with *N*-terminal hexa-histidine tags. For fungal *NAT* ORFs without *Nde*I sites, a more direct approach was used to transfer each insert from the pGEM-5zf(+) to the pET28b(+) vector, involving PCR with primers (Supplementary Table 1) designed to incorporate *Nde*I and *Not*I restriction sites at the beginning and the end of each amplified product, respectively. Ligation products with pET28b(+) vector were initially transformed into the JM109 strain and recombinant colonies were validated by sequencing with vector specific primers (T7-promoter and T7-terminator). The constructs were then transformed into *E. coli* BL21(DE3)pLysS competent cells and clones used for recombinant protein expression were again verified by sequencing with the same primers. Unless otherwise stated, all cloning reagents were from Promega. Sequencing of plasmid DNA was carried out by GATC Biotech or the USDA-ARS Eastern Regional Research Center Integrated Biomolecular Resources (Wyndmoor, PA, USA).

### Recombinant expression-purification of fungal NAT proteins

Recombinant expression of fungal NAT proteins was carried out by modification of the protocol described for human NAT2^18^ [ http://www.thesgc.org/structures/2pfr], and the details are provided in the Supplementary Methods.

### Enzyme activity assays with recombinant proteins

Measurement of enzymatic release of CoA during NAT-catalyzed reactions was performed as described^60^ and the specific details are provided in the Supplementary Methods.

### Enzyme activity assays with cell extracts

After grinding of filtered culture pellets, fungal cell extracts were prepared in buffer (1 ml per 50 ml culture) consisting of 20 mM Tris-HCl (pH 7.5), 1 mM dithiothreitol (added fresh) and 1x protease inhibitors (Thermo/Pearce). Homogenous suspensions were generated through vigorous shaking, and the insoluble and soluble fractions were separated by centrifugation at 40,000 xg (30 min, 4 ^o^C), followed by a second centrifugation (20,000 xg, 40 min, 4 ^o^C) of the supernatant, to eliminate any remaining insoluble material. All manipulations were performed on ice and the supernatants were stored in −80 ^o^C in aliquots that were allowed to thaw only once for immediate use. Quantification of total soluble protein in the preparations was performed against a series of bovine serum albumin standards (0–1 mg/ml), using the Bradford reagent (Sigma-Aldrich). Measurement of enzymatic conversion of arylamine during NAT-catalyzed reactions was subsequently performed as previously described^60^, and the specific details are provided in the Supplementary Methods.

### Differential scanning fluorimetry

DSF was performed essentially as described^60^, each 20 μl reaction containing 3 μg of glycerol-free purified recombinant fungal NAT protein, or protein with 0.4 mM acyl-CoA (acetyl-, n-propionyl-, malonyl- or succinyl-CoA). Reactions were performed in duplicate, with SyproOrange (Invitrogen) in 20 mM Tris-HCl (pH 7.5), 0.5% v/v dimethyl sulphoxide (DMSO). Fluorescence was monitored on a 7500 real-time thermocycler (Applied Biosystems), and the generated sigmoid curves were fitted to the Boltzmann equation to accurately calculate Tm values. Tm peaks of proteins were determined via calculation of the derivative of generated thermal profiles for each set of conditions. Data analysis was performed using software OriginPro 8 SR0 (OriginLab).

### Compounds used in enzyme assays

All compounds were purchased from Sigma-Aldrich. The following acyl-CoA compounds (5 mM in water) were tested in NAT enzymatic activity assays: acetyl-CoA sodium salt, n-propionyl-CoA lithium salt, malonyl-CoA lithium salt, succinyl-CoA sodium salt, butyryl-CoA lithium salt hydrate, 2-butenoyl-CoA lithium salt, glutaryl-CoA lithium salt, hexanoyl-CoA trilithium salt hydrate and octanoyl-CoA lithium salt hydrate. The tested arylamines and arylhydrazines (100 mM in DMSO, except hydralazine which is water-soluble) are described with abbreviations and PubChem ID numbers in the Supplementary Methods.

## Additional Information

**How to cite this article**: Karagianni, E. P. *et al.* Homologues of xenobiotic metabolizing *N*-acetyltransferases in plant-associated fungi: Novel functions for an old enzyme family. *Sci. Rep.* **5**, 12900; doi: 10.1038/srep12900 (2015).

## Supplementary Material

Supplementary Information

## Figures and Tables

**Table 1:** Description of characterized *NAT* loci.

Species (strain)	Taxon mnemonic^1^	Taxon ID^1^	Gene symbol	ORF (bp)^2^	Protein (aa)^2^	Exon span	Number of introns	Predicted locus tag^3^	Nucleotide ID^4^	
*F. verticillioides* (FGSC 7600)	GIBM7	334819	*ΝΑΤ1*	1038	345	1 (1–1038)	0	FVEG_12636	EU552489, FN687904	
			*ΝΑΤ2*	957	318	1 (1–957)	0	FVEG_03961	FN687889, FN687905	
			*ΝΑΤ3*	978	325	1 (1–978)	0	FVEG_12062	FN687890, FN687906	
			*NAT4*^5^	Transcribed pseudogene		1 (1–298) 2 (461–1033)	1	FVEG_07425	FN687891, LN829129	
*F. graminearum* (PH-1)	GIBZE	229533	*ΝΑΤ1*	1032	343	1 (1–1032)	0	FGSG_00080	FN687882, FN687897	
			*ΝΑΤ2*	957	318	1 (1–371) 2 (430–1015)	1	FGSG_09400	FN687883, FN687898	
			*ΝΑΤ3*	960	319	1 (1–960)	0	FGSG_07888	FN687884, FN687899	
*F. oxysporum* f.sp. *lycopersici* (FOL 4287)	FUSO4	426428	*ΝΑΤ1*	1053	350	1 (1–1053)	0	FOXG_15318	FN687885, FN687900	
			*ΝΑΤ2*	957	318	1 (1–371) 2 (423–1008)	1	FOXG_06095	FN687886, FN687901	
			*ΝΑΤ3*	999	332	1 (1–999)	0	FOXG_03795	FN687887, FN687902	
			*ΝΑΤ4*	963	320	1 (1–365) 2 (548–1145)	1	FOXG_04301	FN687888, FN687903	
*A. flavus* (NRRL 3357)	ASPFN	332952	*NAT1*^5^	Elusive	Elusive	Elusive	Elusive	AFL2G_05055	−	
			*ΝΑΤ2*	981	326	1 (1–426) 2 (482–528) 3 (588–1095)	2	AFL2G_01915	FN687893, FN687907	
			*ΝΑΤ3*	957	318	1 (1–395) 2 (449–1010)	1	AFL2G_11316	FN687894, FN687908	
			*ΝΑΤ4*^5^	Transcribed pseudogene		1 (1–91) 2 (143–692)	1	AFL2G_03311	FN687895, FN687909	
*A. nidulans* (FGSC A4)	EMENI	227321	*ΝΑΤ1*	960	319	1 (1–407) 2 (463–510) 3 (568–1072)	2	ANID_10723	FN687881, FN687896	

^1^The taxon mnemonics and ID numbers are from the UniProt Taxonomy database (http://www.uniprot.org/taxonomy/). They correspond to sequenced strains of *Fusarium verticillioides* (teleomorph *Gibberella moniliformis*), *Fusarium graminearum* (teleomorph *Gibberella zeae*), *Fusarium oxysporum* f.sp. *lycopersici*, *Aspergillus flavus* and *Aspergillus nidulans* (teleomorph *Emericella nidulans*). According to current consensus nomenclature guidelines (Supplementary Methods and http://nat.mbg.duth.gr/), taxon mnemonics are attached to the symbols of *NAT* genes to identify their specific organism of origin.

^2^The sequences of open reading frames (ORF) in base pairs (bp), as well as of deduced proteins in amino acids (aa), were determined via alignment of amplification products generated from genomic DNA and cDNA.

^3^The locus tags represent annotations by the Broad Institute (http://www.broadinstitute.org/science/projects/fungal-genome-initiative/gene-finding-methods). *F. verticillioides* FVEG_07425 and *A. flavus* AFL2G_03311 tag genomic loci with sequences overlapping, but not coinciding, with the *NAT* sequences characterized experimentally in the present study.

^4^Two Nucleotide IDs were assigned to each fungal *NAT*, the first for the genomic and the second for the transcribed sequence of each locus.

^5^Annotation remains elusive for the *NAT1* locus of *A. flavus*, as specific amplification from cDNA of the fungus has not been possible. The *NAT4* loci of *F. verticillioides* and *A. flavus* appeared as transcribing pseudogenes with hypothetical ORFs that are disrupted by nonsense mutations.
