# Supplementary material for: Homologues of xenobiotic metabolizing N-acetyltransferases in plant-associated fungi: Novel functions for an old enzyme family
Source: Sci Rep. 2015 Aug 6;5:12900. doi: 10.1038/srep12900 (PMC4542470; doi:10.1038/srep12900)
Supplement: Supplementary Information [file srep12900-s1.pdf]

SUPPLEMENTARY INFORMATION

To manuscript:

Homologues of xenobiotic metabolizing *N*-acetyltransferases in plant-associated fungi: Novel functions for an old enzyme family

Eleni P. Karagianni[1,+], Evanthia Kontomina[1,+], Britton Davis[2], Barbara Kotseli[1], Theodora Tsirka[1], Vasiliki Garefalaki[1], Edith Sim[3], Anthony E. Glenn[2], Sotiria Boukouvala[1]*

[1]Democritus University of Thrace, Department of Molecular Biology and Genetics, Alexandroupolis 68100, Greece.

[2]United States Department of Agriculture, Agricultural Research Service, Toxicology & Mycotoxin Research Unit, Richard B. Russell Research Center, 950 College Station Road, Athens, Georgia 30605, U.S.A.

[3]University of Oxford, Department of Pharmacology, Mansfield Road, Oxford OX1 3QT, U.K.

[+]These authors contributed equally to this work

*Corresponding author: sboukouv@mbg.duth.gr

Supplementary Methods

Nomenclature of fungal NAT genes

Fungal taxonomy has moved away from its dual nomenclature system and the scientific community has now adopted the "one fungus, one name" model. Hence, the consensus anamorph names *Fusarium* and *Aspergillus* have been retained for the five fungi in the present study. However, the teleomorph names are still used by genomic and taxonomy databases, introducing an inevitable confusion to gene nomenclature that is explained below:

According to the consensus guidelines of the Arylamine *N*-acetyltransferase Gene Nomenclature Committee (http://nat.mbg.duth.gr/), the nomenclature of *NAT* genes and proteins is species-specific, meaning that a taxon mnemonic must always precede the *NAT* gene symbol (see NAT website and the latest update of NAT nomenclature by Hein *et al.* (2008), *Pharmacogenet. Genomics* 18: 367-368). Official taxon mnemonics are available from the UniProt Taxonomy database (http://www.uniprot.org/help/taxonomy) and correspond to unique taxon identification numbers (taxon IDs) that must be provided for each species, but not incorporated in the *NAT* gene symbol. For the five fungi of this study, the UniProt Taxonomy database provides the following taxon mnemonics:

- GIBM7 for *Fusarium verticillioides* strain FGSC 7600 (teleomorph *Gibberella moniliformis*)
- GIBZE for *Fusarium graminearum* strain PH-1 (teleomorph *Gibberella zeae*)
- FUSO4 for *Fusarium oxysporum* f.sp. *lycopersici* strain FOL 4287
- ASPFN for *Aspergillus flavus* strain NRRL 3357

- EMENI for *Aspergillus nidulans* strain FGSC A4 (teleomorph *Emericella nidulans*)

It is apparent that UniProt Taxonomy provides taxon mnemonics that are derived from the teleomorph names of *F. verticillioides*, *F. graminearum* and *A. nidulans*. As this is an unavoidable limitation of nomenclature, we try to facilitate the reader of this manuscript by providing both the anamorph and teleomorph names of each fungus in the beginning of the Results and Methods sections, as well as in Table 1 and in all figure legends. We link this nomenclature to the official taxon mnemonics attached to the symbols of NAT homologues.

Recombinant expression-purification of fungal NAT proteins

Frozen stocks (-80 ^{o}C, 10% v/v glycerol) of transformed *E. coli* BL21(DE3)pLysS cells were used to initiate 5 ml overnight cultures (37 ^{o}C, 180 rpm) in Terrific Broth (TB) medium with kanamycin (50 μg/ml). Two millilitres of each starter culture were transferred to 200 ml of TB/kanamycin medium and the cells were grown (37 ^{o}C, 180 rpm) to an optical density (600 nm) of approximately 1.0. Induction took place with 1 mM isopropyl-1-thio-D-galactopyranoside (IPTG) and overnight incubation at 16 ^{o}C on the shaker. Cells were pelleted by centrifugation (6,000 xg, 20 min, 4 ^{o}C) and placed at -80 ^{o}C overnight. After being allowed to thaw slowly on ice, the cell paste was resuspended in 7 ml of lysis buffer {20 mM Tris-HCl pH 7.5, 300 mM NaCl, 5 mM imidazol, 5% v/v glycerol, 0.1% w/v 3-[(3-cholamidopropyl)dimethylammonio]-1-propanesulfonate (CHAPS) and 1x protease inhibitor cocktail from Thermo Scientific}, and subjected to sonication on ice (5-10 cycles of 30 s at 10 kHz with 1-2 min intervals). The soluble and insoluble fractions were separated by centrifugation

(20,000 xg, 40 min, 4 ^{o}C) and the supernatant was immediately subjected to purification of recombinant protein. This was carried out by affinity chromatography through 0.5 ml of Nickel-charged Profinity IMAC resin (BioRad), washed with 5 ml of 20 mM Tris-HCl (pH 7.5), 300 mM NaCl. The soluble fraction of each bacterial lysate was added to the resin and incubated on a shaker for 90 min, prior to elution. Hexa-histidine tagged proteins were recovered from each column with 2.5-5 ml of 20 mM Tris-HCl (pH 7.5), 300 mM NaCl buffer containing increasing (10, 25, 50, 100, 200, 250 mM) concentrations of imidazol. The purification procedure took place at 4 ^{o}C and the recovered fractions were stored at -80 ^{o}C in aliquots with 10% v/v glycerol, that were thawed only once for immediate use in enzymatic activity assays. All NAT proteins were assessed by SDS-PAGE and their calculated molecular weights were in the range of 35.6 to 40.2 kDa. The protein amount was measured by spectrophotometry at 280 nm, before freezing of each aliquot and after thawing and supernatant recovery (20,000 xg, 20 min, 4 ^{o}C) for enzymatic activity assays. Extinction coefficients of recombinant proteins were computed assuming that cysteine residues are either reduced or appear as half cystines, and the average of the two values was used in subsequent calculations of protein concentration. Molecular weights and extinction coefficients of recombinant proteins were calculated with ProtParam (http://web.expasy.org/protparam/). The variable yield and purity of recombinant proteins was not considered as a limitation, since the aim of subsequent activity assays was to investigate the substrate selectivity of each individual enzyme, instead of directly comparing the enzymes to each other. Hence, we preferred to handle recombinant protein preparations in a consistent manner throughout the study, and used the same chromatographic fraction (typically from elution with 50 mM imidazol) of the same protein preparation per set of enzymatic activity assays.

Enzyme activity assays with recombinant proteins

Each 50 µl reaction was initiated by addition of 0.4 mM acyl-CoA to purified recombinant NAT protein (0.5 or 1 µg) and 0.5 mM of substrate in 20 mM Tris-HCl (pH 7.5) buffer. Reactions were terminated after 0-15 min, by addition of 12.5 µl of reagent containing 5 mM 5,5′-dithiobis-2-nitrobenzoic acid (Ellman's reagent) and 6.4 M guanidine-HCl in 0.1 M Tris-HCl (pH 7.5). The absorbance of the coloured product was quicky measured at 405 nm using a TECAN M1000 microplate reader. Assays were performed in duplicate and control reactions, lacking recombinant NAT protein, where performed to assess possible spontaneous hydrolysis of acyl-CoA compounds during the reaction. Endogenous NAT activity in *E. coli* is below detectable levels when affinity chromatography purified preparations of recombinant proteins are used in assays. The amount (nmol) of produced CoA was determined from linear (R^2 >99.5%) plots of OD (405 nm) against a series of standards containing 0-17.5 nmol of CoA and 0.5 mM substrate. Enzyme specific activity was determined as nmol of produced CoA per min per mg of recombinant protein.

Enzyme activity assays with cell extracts

Enzyme activity assays were performed in 100 µl reactions containing 80-110 µg of total soluble protein, 0.1 mM of 3,4DCA and 0.4 mM of acetyl-CoA or malonyl-CoA in 20 mM Tris-HCl (pH 7.5), 1 mM dithiothreitol (added fresh). After specific incubation times (typically 0-12 min, or up to 30 min), reactions were terminated with 20% w/v trichloroacetic acid. Following addition of 5% w/v *p*-

dimethylaminobenzaldehyde in 9:1 v/v acetonitrile:water (Erlich's reagent), absorbance was measured at 450 nm using a conventional Beckman Coulter DU 730 spectrophotometer. All reactions were performed in triplicate and a no lysate control was always included to assess the inherent stability of the arylamine under the assay conditions applied (for example, 3,4DCA was preferred over 2AP, as it was determined to be considerably more stable during prolonged incubation periods). The amount (nmol) of substrate was determined from linear (R^2 >99.9%) plots of OD (450 nm) against a series of 3,4DCA standards (0-30 nmol). Enzyme specific activity was determined as nmol of consumed arylamine per min per mg of total protein.

Acceptor substrates used in enzyme assays

The following arylamines and arylhydrazines were used in the study (abbreviations and PubChem ID numbers in parentheses): 2-aminophenol (2AP; CID 5801), 4-chloroaniline (CLA; CID 7812), 4-bromoaniline (BRA; CID 7807), 4-iodoaniline (IOA; CID 10893), 4-anisidine (ANS; CID 7732), 4-phenoxyaniline (POA; CID 8764), 4-aminobenzoate (PABA; CID 4876), 5-aminosalicylate (5AS; CID 4075), 4-aminosalicylate (4AS; CID 4649), 2,4-dichloroaniline (2,4DCA; CID 11123), 3,4-dichloroaniline (3,4DCA; CID 7257), 4-aminoveratrole (AMV; CID 22770), sulphamethazine (SMZ; CID 5327), procainamide (PA; CID 4913), 4-aminobenzoylglutamate (PABG; CID 196473), 4-aminopyridine (APY; CID 1727), isoniazid (INH; CID 3767) and hydralazine (HDZ; CID 3637). For use in culture media, 2-benzoxazolinone (BOA; CID 6043) and 3,4DCA were dissolved in ethanol to 40 mg/ml.

Supplementary Figure legends

Supplementary Fig. S1: Alignment of deduced amino acid sequences of characterized fungal NAT proteins. Amino acid identities/similarities are shaded with red/grey colour. Blue horizontal lines indicate semi-conserved motifs characteristic of NAT enzymes. The yellow dots indicate the cysteine (C) - histidine (H) - aspartate (D) residues forming the conserved catalytic triad. The alignment was constructed with BioEdit Sequence Alignment Editor 7.0.5.3, using the amino acid sequences deduced from the transcribed (intronless) sequences of Table 1 for all 13 NAT proteins expressed in recombinant form. UniProt Taxonomy organism-specific mnemonics ASPFN, EMENI, FUSO4, GIBM7 and GIBZE are used as prefixes to the symbols of NAT proteins of sequenced strains of *A. flavus*, *A. nidulans* (*E. nidulans*), *F. oxysporum* f.sp. *lycopersici*, *F. verticillioides* (*G. moniliformis*) and *F. graminearum* (*G. zeae*), respectively.

Supplementary Fig. S2: Recombinant fungal NAT proteins purified by affinity chromatography and visualized by SDS-PAGE/Coomassie blue staining. Chromatographic fractions eluted with 0, 10, 25, 50, 100, 200 and 250 mM of imidazol are shown in lanes 1-7, respectively. Asterisks indicate lanes with bacterial soluble extracts prior to purification. Lanes M are Precision Plus Protein™ Standards from Bio-Rad, with the 37 kDa marker indicating the approximate size of NAT protein bands (also indicated by arrows). **a-c**: NAT1, 2 and 3 of *F. verticillioides* (*G. moniliformis*); **d-f**: NAT1, 2 and 3 of *F. graminearum* (*G. zeae*); **g-j**: NAT1, 2, 3 and 4 of *F. oxysporum* f.sp. *lycopersici*; **k,l**: NAT2 and 3 of *A. flavus*; **m**: NAT1 of *A. nidulans* (*E. nidulans*). Recombinant proteins eluted with 50 or 100 mM imidazol (lanes 4 or 5) were typically used in enzymatic analyses.

Supplementary Fig. S3: Fungal NAT proteins assayed for enzymatic activity with different acyl-coenzyme A compounds. Ellman's reagent was used to monitor CoA production in enzymatic reactions with 1 µg of recombinant protein, 0.5 mM of 5-aminosalicylate and 0.4 mM of acyl-CoA. Each data point is the average value of two replicates ± standard deviation. **a-c**: NAT1, 2 and 3 of *F. verticillioides* (*G. moniliformis* - GIBM7)*;* **d-f**: NAT1, 2 and 3 of *F. graminearum* (*G. zeae* - GIBZE); **g-j**: NAT1, 2, 3 and 4 of *F. oxysporum* f.sp. *lycopersici* (FUSO4); **k,l**: NAT2 and 3 of *A. flavus* (ASPFN); **m**: NAT1 of *A. nidulans* (*E. nidulans* -EMENI).

Supplementary Fig. S4: Fungal NAT proteins assayed by differential scanning fluorimetry in the presence of various acyl-coenzyme A compounds. DSF was used to monitor changes in thermal stability of recombinant proteins upon selective acyl-CoA binding. The panel on the left shows the change in SyproOrange fluorescence upon temperature increase. The panel on the right shows the derivative of generated thermal profiles, with main peaks demonstrating protein Tm values. In both panels, the arbitrary units on the *y*-axis are in billion. Two replicate experiments were performed, generating overlapping curves for which the average plot is shown. **a-c**: NAT1, 2 and 3 of *F. verticillioides* (*G. moniliformis* - GIBM7)*;* **d-f**: NAT1, 2 and 3 of *F. graminearum* (*G. zeae* - GIBZE); **g-i**: NAT1, 2, and 4 of *F. oxysporum* f.sp. *lycopersici* (FUSO4); **j,k**: NAT2 and 3 of *A. flavus* (ASPFN).

Supplementary Fig. S5: Fungal NAT proteins assayed for enzymatic activity with different acceptor substrates. Ellman's reagent was used to monitor CoA production in enzymatic reactions containing a fixed amount (0.5 or 1 μg) of recombinant protein, 0.4 mM of selective acyl-CoA and 0.5 mM of substrate. Each data point is the average value of two replicates ± standard deviation. **a-c**: NAT1 and 3 of *F. verticillioides* (*G. moniliformis* - GIBM7), the former assayed with malonyl-CoA (a) and the latter with acetyl-CoA (b) and n-propionyl-CoA (c)*;* **d-f**: NAT1 and 3 of *F. graminearum* (*G. zeae* - GIBZE), the former assayed with malonyl-CoA (d) and the latter with acetyl-CoA (e) and n-propionyl-CoA (f); **g-i**: NAT1 and 3 of *F. oxysporum* f.sp. *lycopersici* (FUSO4), the former assayed with malonyl-CoA (g) and the latter with acetyl-CoA (h) and n-propionyl-CoA (i); **j-m**: NAT2 and 3 of *A. flavus* (ASPFN), the former assayed with acetyl-CoA (j) and n-propionyl-CoA (k), and the latter with malonyl-CoA (l) and succinyl-CoA (m); **n,o**: NAT1 of *A. nidulans* (*E. nidulans* - EMENI), assayed with acetyl-CoA (n) and n-propionyl-CoA (o). The screen in (a) included the whole panel of substrates. Screens in (b-g), (n) and (o) excluded IOA, APY, PABG, 2,4DCA and BRA. Screens (h-m) additionally excluded AMV. The full chemical names of compounds are: 2-aminophenol (2AP), 4-iodoaniline (IOA), 4-phenoxyaniline (POA), 4-chloroaniline (CLA), 3,4-dichloroaniline (3,4DCA), 4-aminopyridine (APY), isoniazid (INH), hydralazine (HDZ), sulphamethazine (SMZ), 4-aminobenzoylglutamate (PABG), 2,4-dichloroaniline (2,4DCA), 4-aminosalicylate (4AS), 4-anisidine (ANS), 4-aminoveratrole (AMV), 4-bromoaniline (BRA), procainamide (PA), and 4-aminobenzoate (PABA).

FIG. S1

```
                     10         20         30         40         50         60         70         80
(ASPFN)NAT2  ---------- -----MASPS QIYNSEQLEL YLERIGYADS VNATLDNTTG RLDHVLQSIQ QDRLATLTKL QRRHLASIPW
(ASPFN)NAT3  ---------- --------MS SAYSALQITK YLSYLSLPA- --------KY HAYVETPHLF PKDEAALTVL FRCQITRVPF
(EMENI)NAT1  ---------- -----MAS-- -TFTRSQLEA YLQRIGYANS ASG---PECP RLHQLQASIE QDALKALEEL QRRHISSIPW
(FUSO4)NAT1  MARLEDPTAL TQLPLEETAR VRYSPSELQD YFKTIKLAKR FLDLG--NSV LKDAALARTK EHGLPLLQAI TRYHTCNVPF
(FUSO4)NAT2  ---------- --------MS AIYSEAQVAG FLKHLQIPQ- --------EF YVGNE-PILD ---HAFLKVL HQHMIATVPY
(FUSO4)NAT3  ---MSGTRIN FSGNCNMADR IRYSRSQLEK YYDRIAFPA- --------SD RQYDISNLSS EDQQSYLDTL TKQQILTVPF
(FUSO4)NAT4  ---------- --------MT SAYSQEQLSQ FLEHINLPK- --------AL REDIQ-PSL- ----MLLKAL HTHSLATFPY
(GIBM7)NAT1  MARLEDPTAL TQLP-DESAR VRYTSSELQD YFETLKFPQR FLDLG--NSV LKDPSLARTK ENGLPLLQAI TRYHTCNVPF
(GIBM7)NAT2  ---------- --------MS AIYSEAQVAG FLKHLQIPQ- --------EF YVGNE-AILD ---HAFLKVL HQHMIATVPY
(GIBM7)NAT3  ---------- ------MADR IRYSKSQLEK YYDRIAFPA- --------SD RRYDISNLSS EDQRSYLDTL TKQQILTVPF
(GIBZE)NAT1  MSCLPDPTVL TQLQVENPP- -RYTQAQLQE YLKVIKLPQR FLDS----PV LKDSTLAHTK EHGLPLIKAI TRYHTCNIPF
(GIBZE)NAT2  ---------- --------MS AIYSEAQVAK FLKHICVPQ- --------EF YVGNE-PILD ---HAFLTVL HQHMIARVPY
(GIBZE)NAT3  ---------- ------MMER IKYSESQLET YYSRINFQD- --------SD PKYTIDNLST EAQIDFLFEL TKRQLLAVPF

                     90        100        110        120        130        140        150        160
(ASPFN)NAT2  GNSALHYSQH QSISTHPACI FDKLVVRRL- -----DGYCM ENTNLFYMVL RCLGYNVYPT GGFVSQAVAG GNQTP--GSE
(ASPFN)NAT3  ENLSVYYSAT RQPDIHPETL YSKMMGAEET GPTGRGGYCL EVNIFFHHIL RGLGFDVYTV GARNRDFVNG VPQGD-----
(EMENI)NAT1  GNSAIHYSQH HSISTYPSAV FEKLVVRRL- -----DGYCM ENTNLLYVVL RSLGYQAYPA AGFVSNAAAD PENAG--SEV
(FUSO4)NAT1  ENLVLHYDPR KIVTLDPAEL YTRIVTRRR- -----GGRCM ENNIFLGTAL RSLGYEVRNC GGFVSRAMSP YPEVRKNQSA
(FUSO4)NAT2  DNLTLHYSSH RNITLEPQAL YQKIVVDGR- ---GRGGYCM ESNLFFCYML PALGFQVYEV GVFVRLRKDG VPHGG-----
(FUSO4)NAT3  ENLTLHYSWH RIVDVNADHL YDRIVNERR- -----GGYCM ENNTLFNTVL LSLGFHTYMV GSR----VFN PDAGR-----
(FUSO4)NAT4  ENLSLHYNAT HSIDLDPQHL FRRMVTDKR- ---GRGGFCM EIAILYNHIL PAIGFDAYTA GVRTRGRLEG VPTGD-----
(GIBM7)NAT1  ENLVLHYDPH KIVTLDPAEL YTRIVTRRR- -----GGRCM ENNIFLGTAL RSLGYEVRNC GGFVSRAMSP YPEVRKNQSA
(GIBM7)NAT2  DNLTLHYSSH RNITLEPQAL YQRIVGDGR- ---GRGGYCM ESNLFFCYML PALGFQVYEV GVFVRLRNNG IPFGG-----
(GIBM7)NAT3  ENLTLHYSWH RIVDVNADHL YDRIVNERR- -----GGYCM ENNTFFNTVL LSLGYHTYMV GSR----VFN PDADR-----
(GIBZE)NAT1  ENLELHYSAH KTITLDPYEL FEKIVTRRR- -----GGRCM ENNTFLGTVL RSFGYEVRDC GGFVARAMSP YPDVRKNQAY
(GIBZE)NAT2  ENMTLHYSSH RNVILEPQAL YQRIVVDGR- ---GRGGYCM ESNLFFCYML PALGFQVYEV GVFVRLRKDG VPHGG-----
(GIBZE)NAT3  ENLTLHYSWH RIVDVNADHL FTKIVTEKR- -----GGYCM ENNSFFHTVL VSLGFEVYMV AAR----VFS PDAKR-----

                    170        180        190        200        210        220        230        240
(ASPFN)NAT2  LYMSLGHMVL IVIIDDQR-Y MVDVGFGNFG PTSPLPLKED GAVAVCMAPA EMRLVKDTPI EFID----RS QKLWIYQIRY
(ASPFN)NAT3  -YGGWVHMAN IVRLPSGVRY HLDVGFGGDG PTRPIPLVSG -ASVQNLGTQ EARLLYDNIS KESQ--RKQ- -NEWIYQCRN
(EMENI)NAT1  RYGSLGHMVI IVGISNQK-Y MVDVGFGNNG PTSPLPLLRN -VSGDLIPPA QMRLDKTPLP EAVD----QS QEEWVYSVRY
(FUSO4)NAT1  TYDGWNHMLL LVLLGDEW-Y GVDVGMGSMG PNLPFPLQDG -FETLSIAPR EIRIQKRSIP ETYATDPSHG TRMWCYDVCY
(FUSO4)NAT2  -YPGWVHIVN IVTLPDNSFW VMDTSFGGDG PTQPMPLAEG -AEWRNLGTQ DARLIKDFIP GQTE--LTSG RRLWIYQCRN
(FUSO4)NAT3  -FGGTSHCLS LVNIAGRT-Y AVDVGFGARN PTEPLEVEHE -RIHKGSSGF QMRLRHDIIA QNVS--NQK- --LWIYEYRS
(FUSO4)NAT4  -YPGWNHIVN IITFPDDSKY HSDVAFGGDG ATMPMPLIDD -LVHENLGTQ QIRLKRDWIP HQVH--RTKE TRLWIYQYRN
(GIBM7)NAT1  TYDGWNHMLL LVFLGDEW-Y GVDVGMGSMG PNLPFPLQDG -FESLSIAPR EIRIQKRSIS ETHATGPSHA TRMWCYDVCY
(GIBM7)NAT2  -YPGWVHIVN IVTLPDNSFW VMDASFGGDG PTQPMPLVEG -AEWHNMGTQ TARLIKDFIP GQTE--LTSG RRLWIYQCRN
(GIBM7)NAT3  -FGGTSHCLS LVIIDGRT-L AVDVGFGGRN PTEPLEVEHE -RVHTGSSGF QMRLRYDAIA QNVS--NQK- --LWIYEYRS
(GIBZE)NAT1  TYDGWNHMLN LAHFEDEW-Y IVDVGMGSMG PNLPFPLRHG -FETTSIAPR KIRLQRRAIA ESHASNPTKG TKLWCYDVCL
(GIBZE)NAT2  -YPGWVHIVN IVTLPDNSFW VIDASFGGDG PTRPMPLIEG -AEWRNMGTQ DARLIKDFIP GQTE--LTSG RRLWIYQCRN
(GIBZE)NAT3  -YGGITHCLN VVTIGDKS-Y AVDVGFGGRT PTIPMEILDG -EVFRRTDSG QMRLRHDTIP EYLT--KQK- --VWIYEFRS

                    250        260        270        280        290        300        310        320
(ASPFN)NAT2  NPESN----W IPQYSFSE-V EFLPQDFAAM NYSTSHRPTS WFVQALVCTR VIMDETGIE- ---------- --------PV
(ASPFN)NAT3  GVDKE----W NSFYCYPD-L EFFQEDFEVI NRFA--AWEF LKRDLIVTVK FIRSGEEGEI LQHQETFVHI PDGPDEVHIA
(EMENI)NAT1  GPDKD----W APMYAFAE-T EFLPQDFAMM NFNTSKGSSS WFTQRVVCVR HILGDDESS- ---------- --------IK
(FUSO4)NAT1  NPAEN-EKIW TPVYCFTE-T EFLPQDYEVM SWFTSTNPKS FFTRYITCTK MIMDEDKEV- ---------- --------II
(FUSO4)NAT2  SPDQP----W ISFYAFSHSV EWLPADFEIS NCYTGTSPRS FQTTTVLIVK FLLR--ESKT SPTGE----- -------EIY
(FUSO4)NAT3  HDGGE----W VPQWCFMD-Y EVLPEDIRVF NMSPSKSPSS FFTFKVVSVQ FTSEKEDYSD GSARNLKNVG G------VID
(FUSO4)NAT4  GQDKE----W NSFYSFPG-I EFFALDWGVV NWWINTHADS HQLRNVLTIK FLLRPVESEA SFEGEM---- -------EIY
(GIBM7)NAT1  NPAES-KKTW TPVYCFTE-T EFLPQDYEVM SWFTSTNPRS FFTRYITCTK MIMDEDKEV- ---------- --------II
(GIBM7)NAT2  SPDLP----W TSFYAFSHSV EWLPADFEIT NCYTGTSPRS FQTSTVLIVK FLLR--ESKT SPTGE----- -------EIY
(GIBM7)NAT3  RDGAE----W VPQWCFMD-F EVLPEDIRVF NLSPSKSPSS FFTFKVVSVQ FTSEKEDYSD GSARDLNNVG G------DVD
(GIBZE)NAT1  KPTEDGTDVW TPTYAFTE-T EFLPQDYEVM SWFTSTNPRS FFTRYVTCTK MIQDEEKEE- ---------- --------II
(GIBZE)NAT2  SPDQN----W NSFYSFSHSV EWLPADFEIA NCYTGTSHRS FQTHTVLVVK FMLR--ESKT STTGE----- -------EIY
(GIBZE)NAT3  NESGE----W IPQWCFID-H EVLPDDIRVM NMAPSKSPSS FFTFKVIAVQ FVSEKEDCSD METRDLKNVD G------VID

                    330        340        350        360        370        380
(ASPFN)NAT2  GIYILSGKEV KKRLRGETET VAIFEKEEDR VNALARWFDM HFLEHEIEGV RGLVSQIK-- ----
(ASPFN)NAT3  GKIMLVNNEV KLNTGGKTKV IETLDTEAAR MKALRKWFSI CLD------- ---------- ----
(EMENI)NAT1  GLYVMAGKQV KRRVHGQTEI VQTLENENDR VEALSKWFGI HLLDHEVAGI QGLVSELK-- ----
(FUSO4)NAT1  GNLTLFKDTV RETIGSGRKV VKKFETEEER IEGLVGIFDV NLTEEEKNSL PQEKRLGQSK V---
(FUSO4)NAT2  GKFMLVNDVV KENPGGKTKV LKELKTEDER VEALKEYFGI DLTTEEREAI KGFQTEIKTQ ----
(FUSO4)NAT3  GAFIIDGNLF KYRKGGETKW EKTFKTEEER LEALWKYFGV ELTKENEFAI RGTAGAIP-- ----
(FUSO4)NAT4  GKFMLVNGVV KENLGGKTQI ITTCNTEGER VEALEKYFQI SLTNEEKEDI HGYVSELRGT MP--
(GIBM7)NAT1  GNLTLFKDTV RETIGSDRKV VKKFETEEER IKGLVEIFDV NLTEEEKNSL PQEKRLA--- ----
(GIBM7)NAT2  GKFMLINDVV KENPGGKTKV LKELKTEDER VEALKEYFDI DLTTEEREAI KGFQTEIKSE ----
(GIBM7)NAT3  GAFVIDGELF KYRKGGETKW ERTFKSEDER LAALRKYYGV EVTKENEFAI GGTAGAISYR RTGS
(GIBZE)NAT1  GNLTLFKDTI RETIGNQRKV IRECKTEEER IQALVEVFDV NLTEEEKNGI PEERRLA--- ----
(GIBZE)NAT2  GKFMLINDVV KENPGGKTIV LKELKTEDER VEALKEYFGI DLTTEEREAI KGFQTEIKSE ----
(GIBZE)NAT3  GSVFIDGNVM KYRKAGVVKM EKTFETEDER LEALKRYFDI ELTDENKFAI RGTAGAIP-- ----
```

FIG. S2

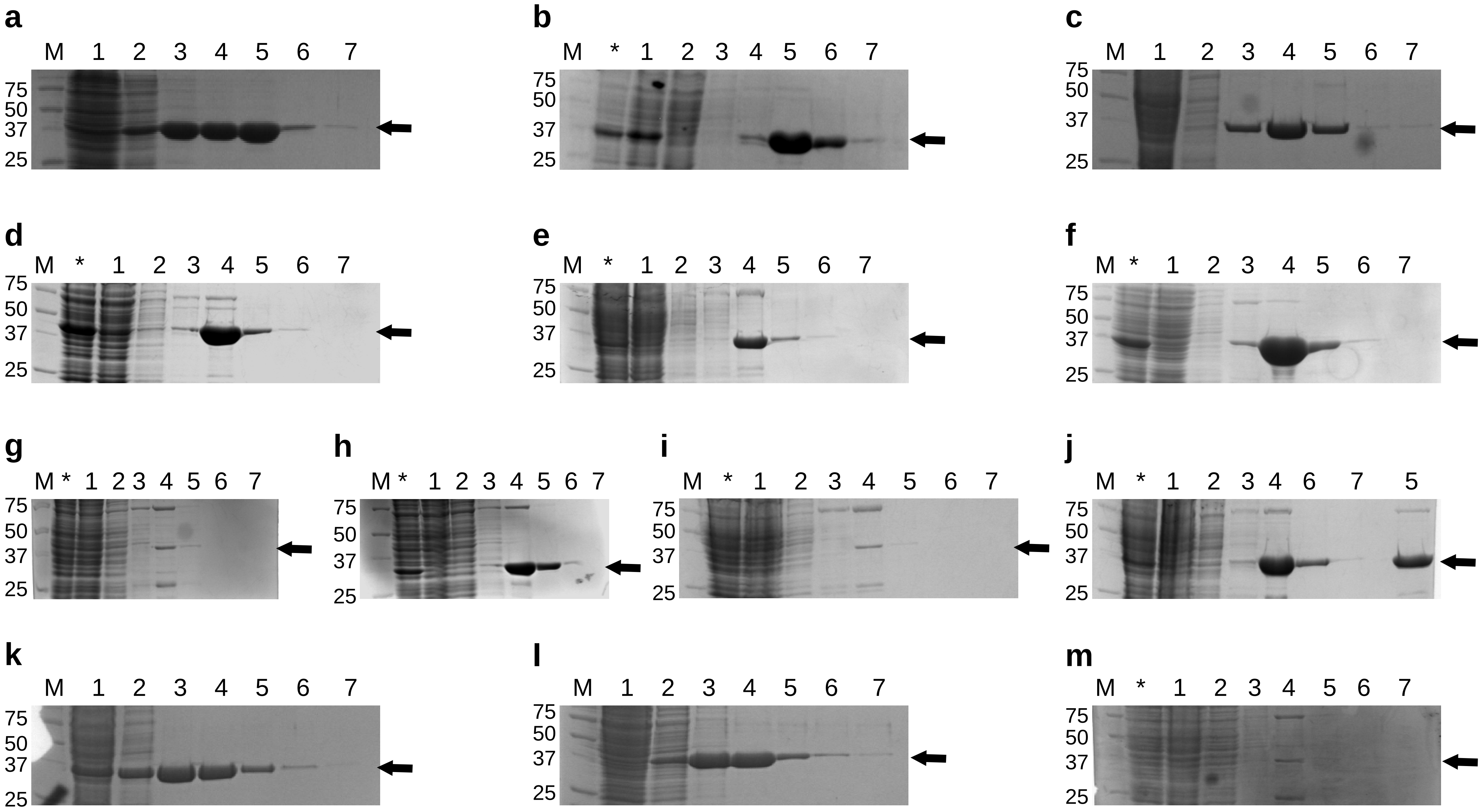
a
M 1 2 3 4 5 6 7
75 50 37 25
b
M * 1 2 3 4 5 6 7
75 50 37 25
c
M 1 2 3 4 5 6 7
75 50 37 25
d
M * 1 2 3 4 5 6 7
75 50 37 25
e
M * 1 2 3 4 5 6 7
75 50 37 25
f
M * 1 2 3 4 5 6 7
75 50 37 25
g
M * 1 2 3 4 5 6 7
75 50 37 25
h
M * 1 2 3 4 5 6 7
75 50 37 25
i
M * 1 2 3 4 5 6 7
75 50 37 25
j
M * 1 2 3 4 6 7 5
75 50 37 25
k
M 1 2 3 4 5 6 7
75 50 37 25
l
M 1 2 3 4 5 6 7
75 50 37 25
m
M * 1 2 3 4 5 6 7
75 50 37 25

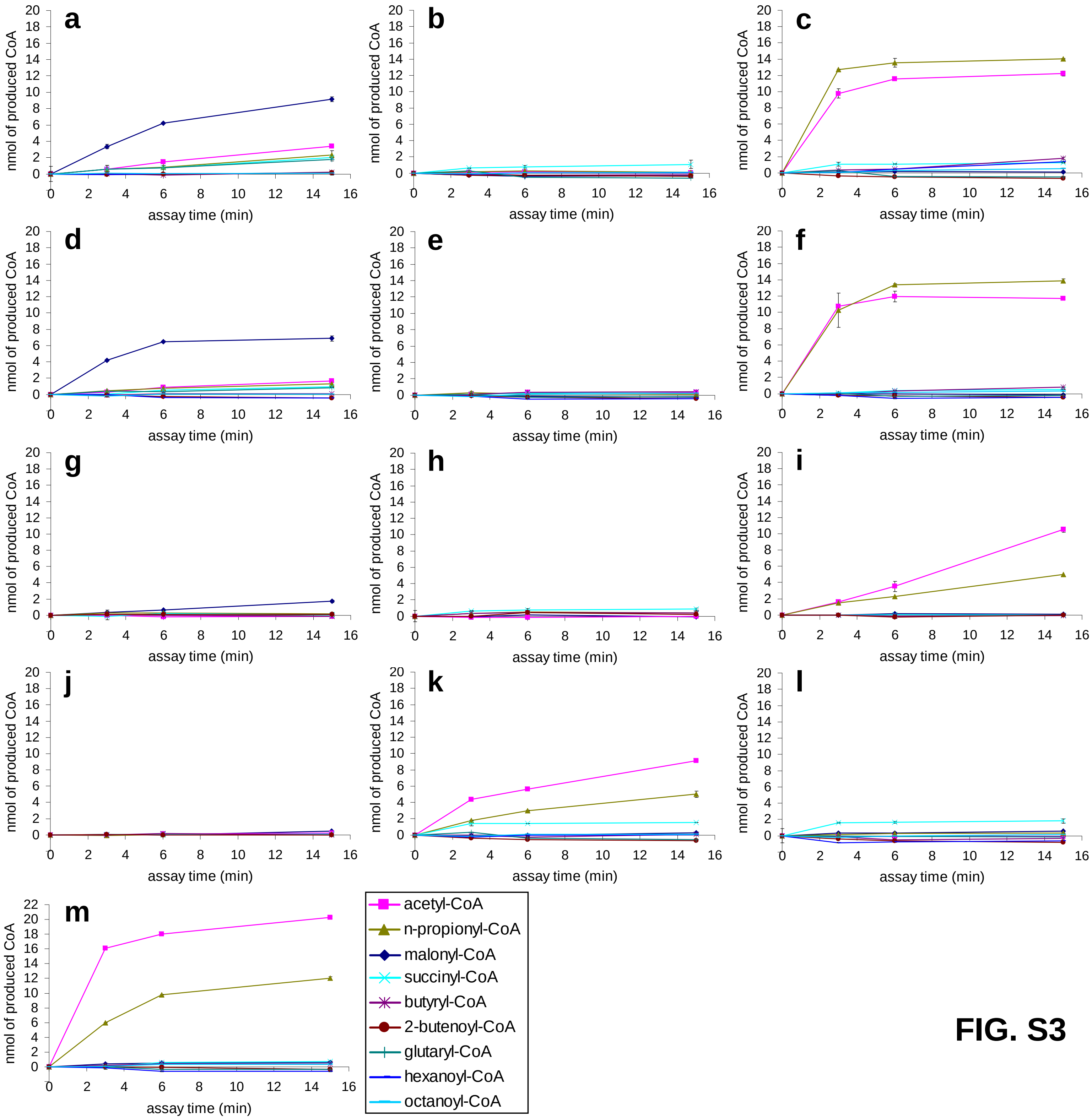

a
b
c
d
e
f
g
h
i
j
k
l
m
nmol of produced CoA
assay time (min)
acetyl-CoA
n-propionyl-CoA
malonyl-CoA
succinyl-CoA
butyryl-CoA
2-butenoyl-CoA
glutaryl-CoA
hexanoyl-CoA
octanoyl-CoA

FIG. S3

FIG. S4

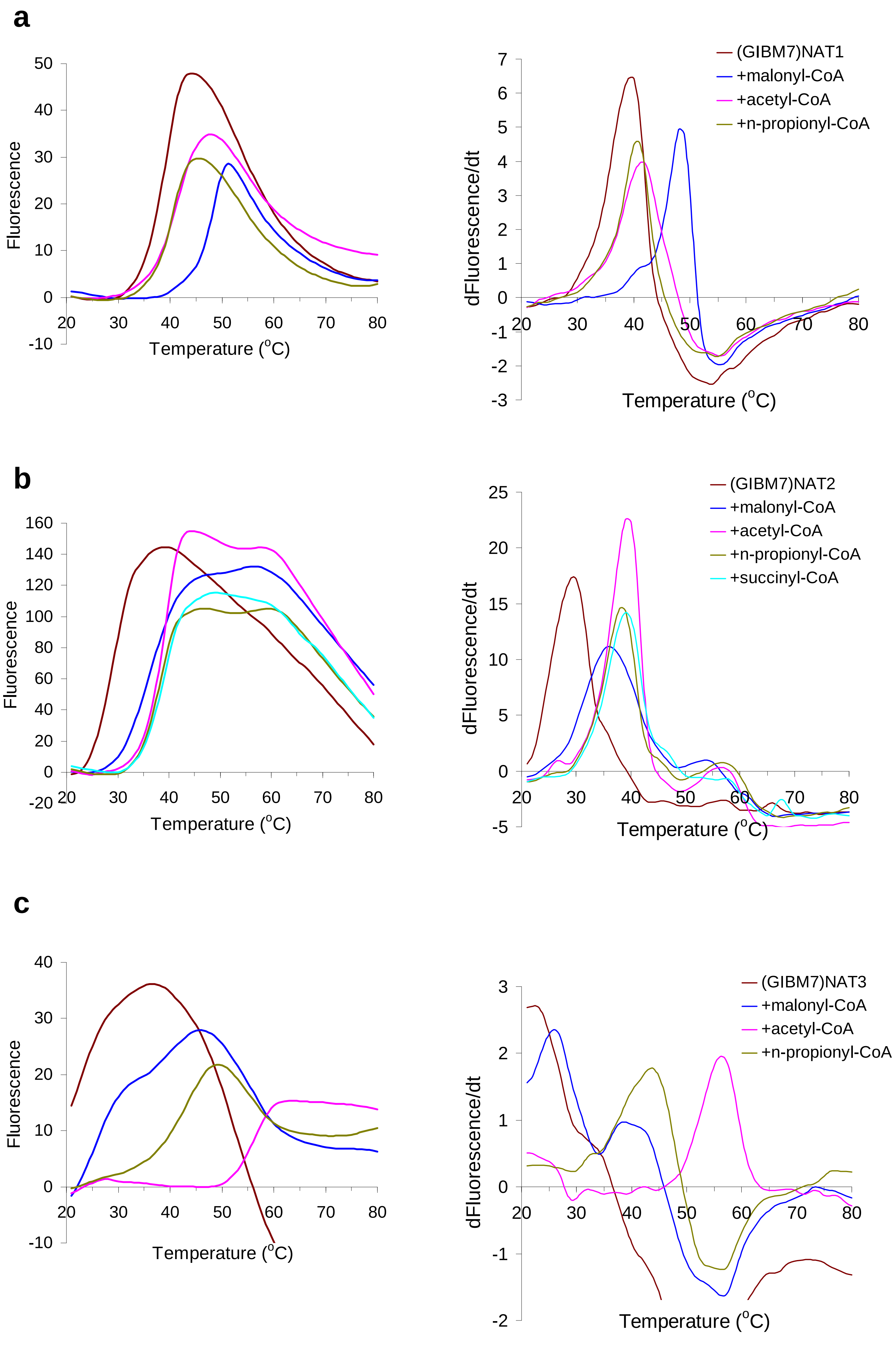
a
Fluorescence
Temperature (°C)
dFluorescence/dt
Temperature (°C)
(GIBM7)NAT1
+malonyl-CoA
+acetyl-CoA
+n-propionyl-CoA
b
Fluorescence
Temperature (°C)
dFluorescence/dt
Temperature (°C)
(GIBM7)NAT2
+malonyl-CoA
+acetyl-CoA
+n-propionyl-CoA
+succinyl-CoA
c
Fluorescence
Temperature (°C)
dFluorescence/dt
Temperature (°C)
(GIBM7)NAT3
+malonyl-CoA
+acetyl-CoA
+n-propionyl-CoA

FIG. S4, contd.

d

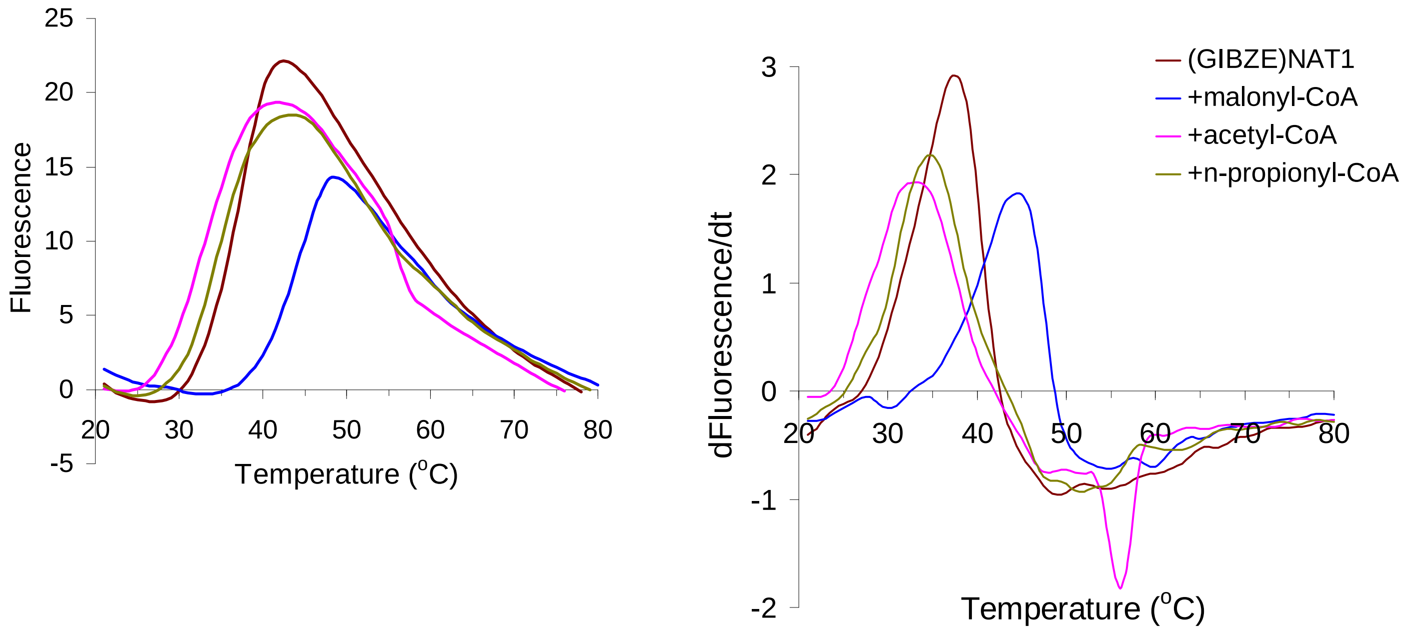

e

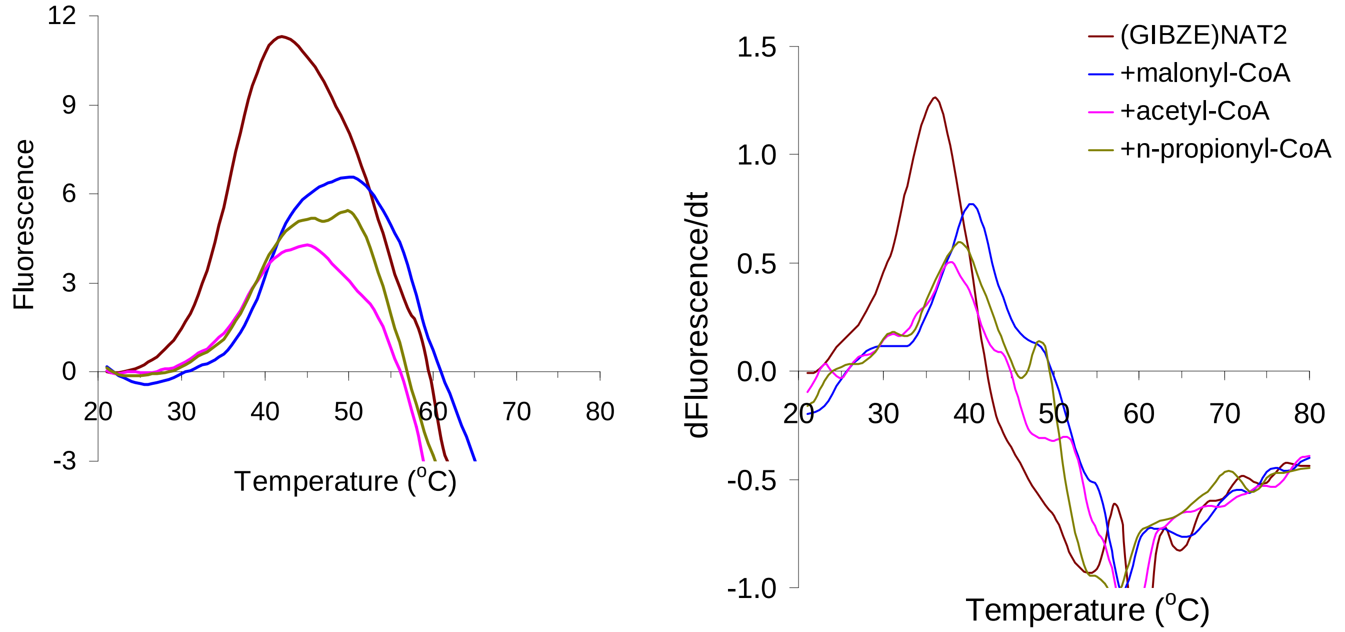

f

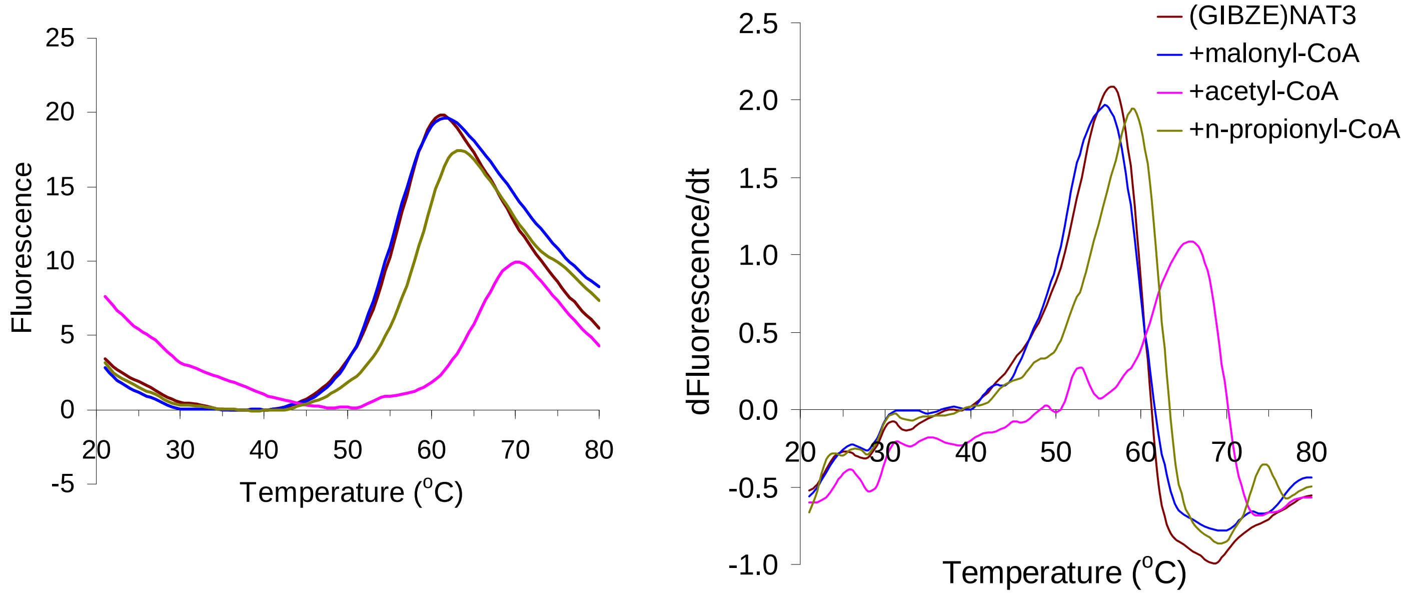

FIG. S4, contd.

g

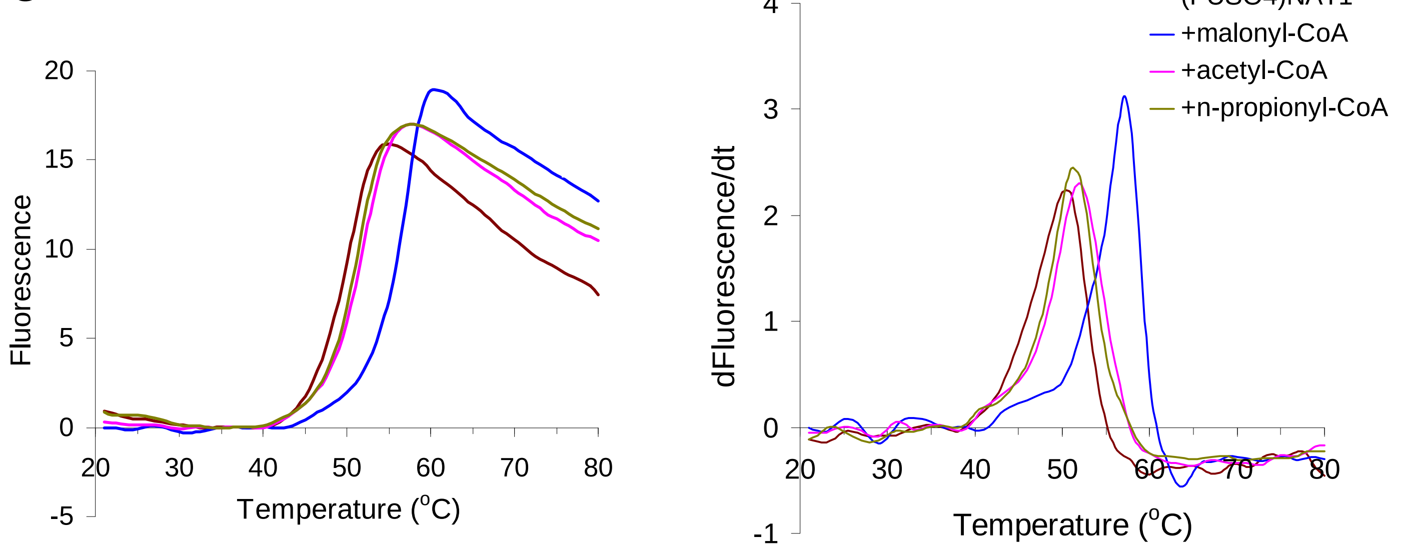

h

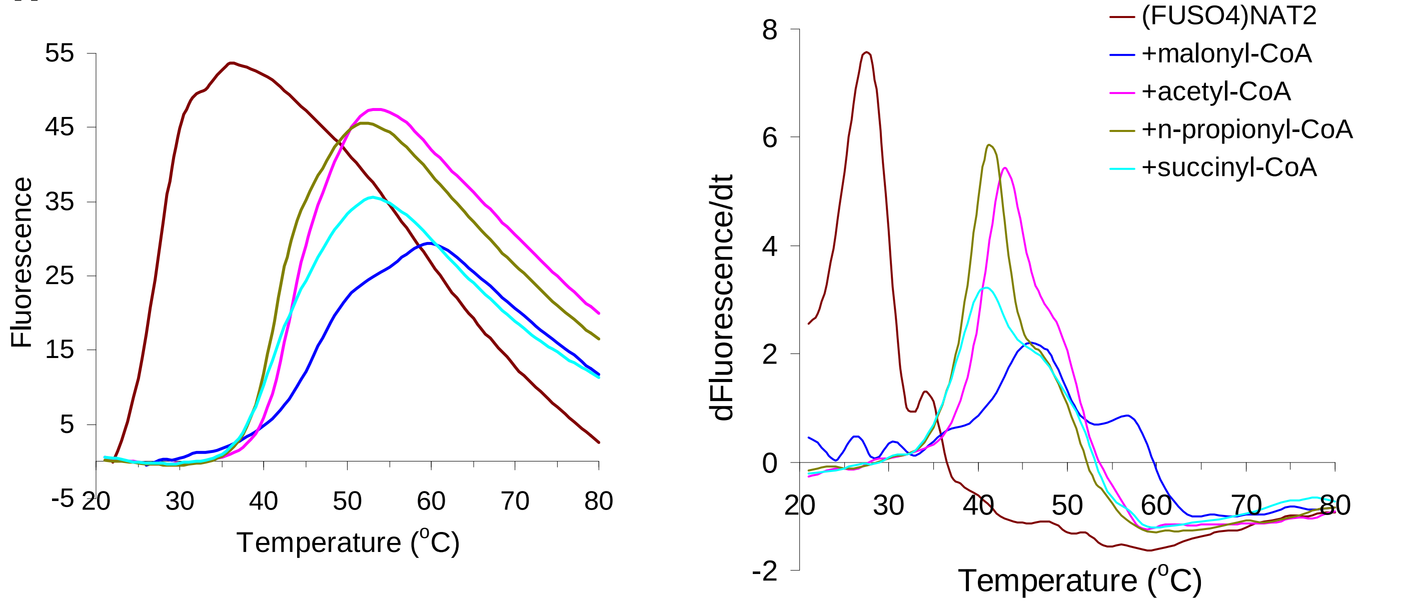

i

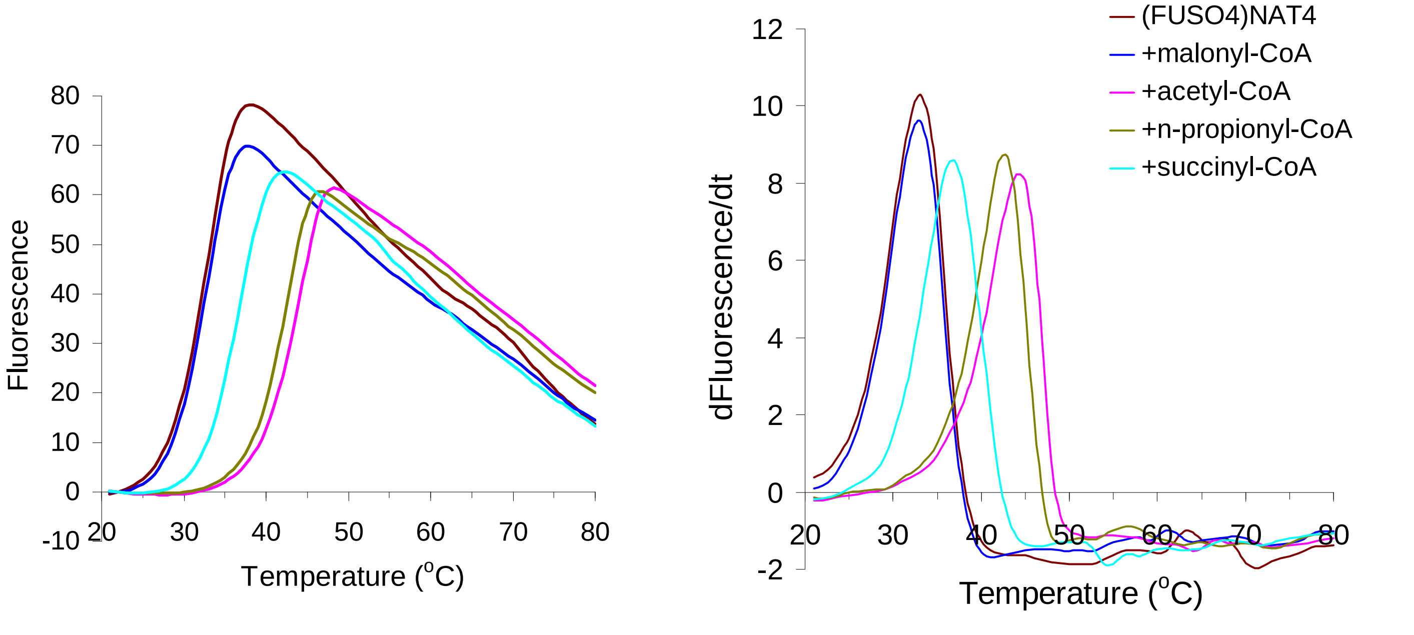

FIG. S4, contd.

j

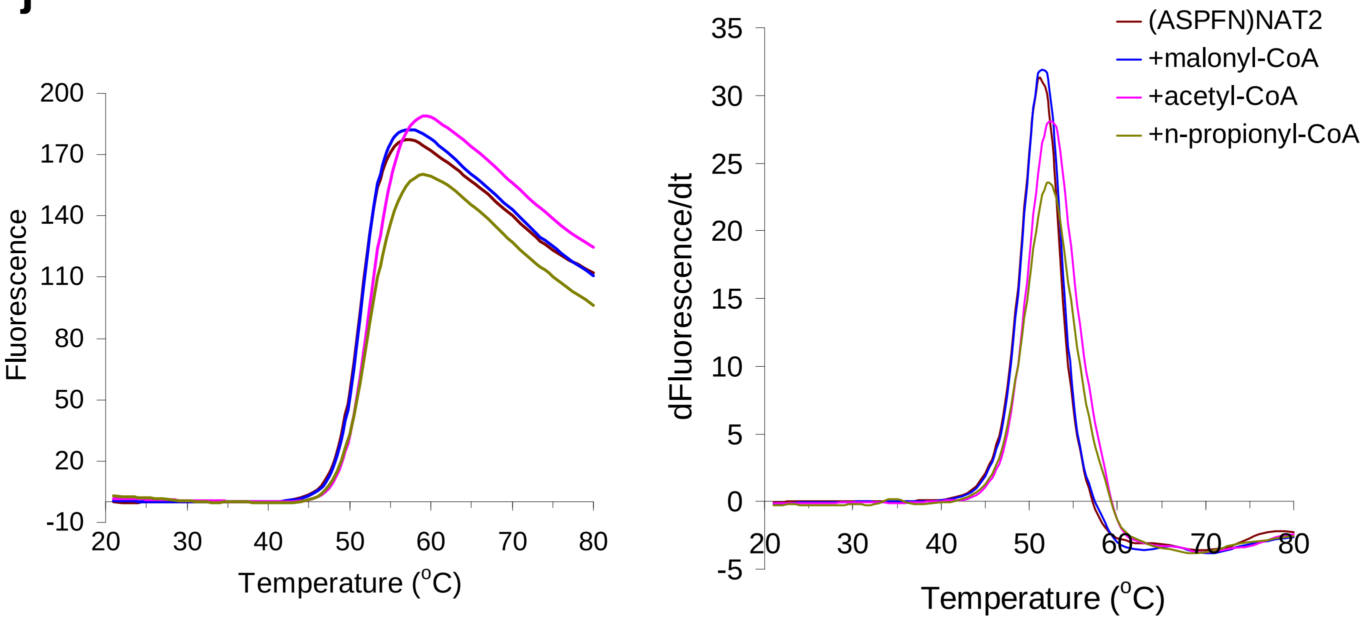

k

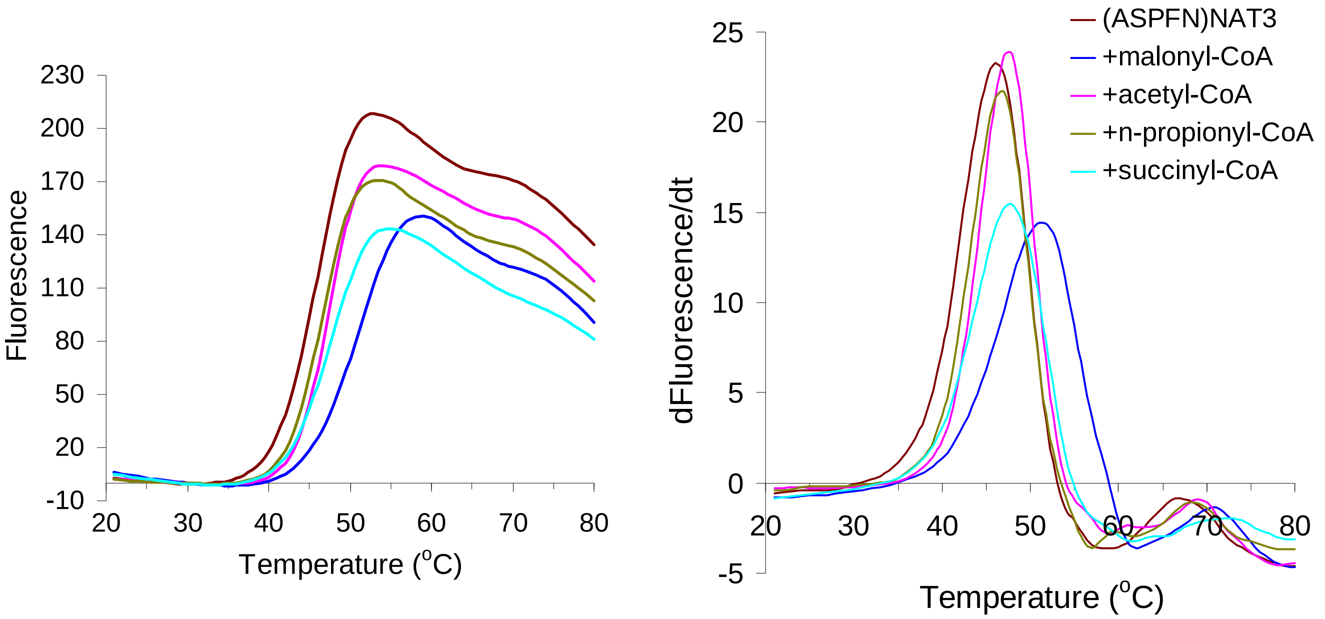

FIG. S5

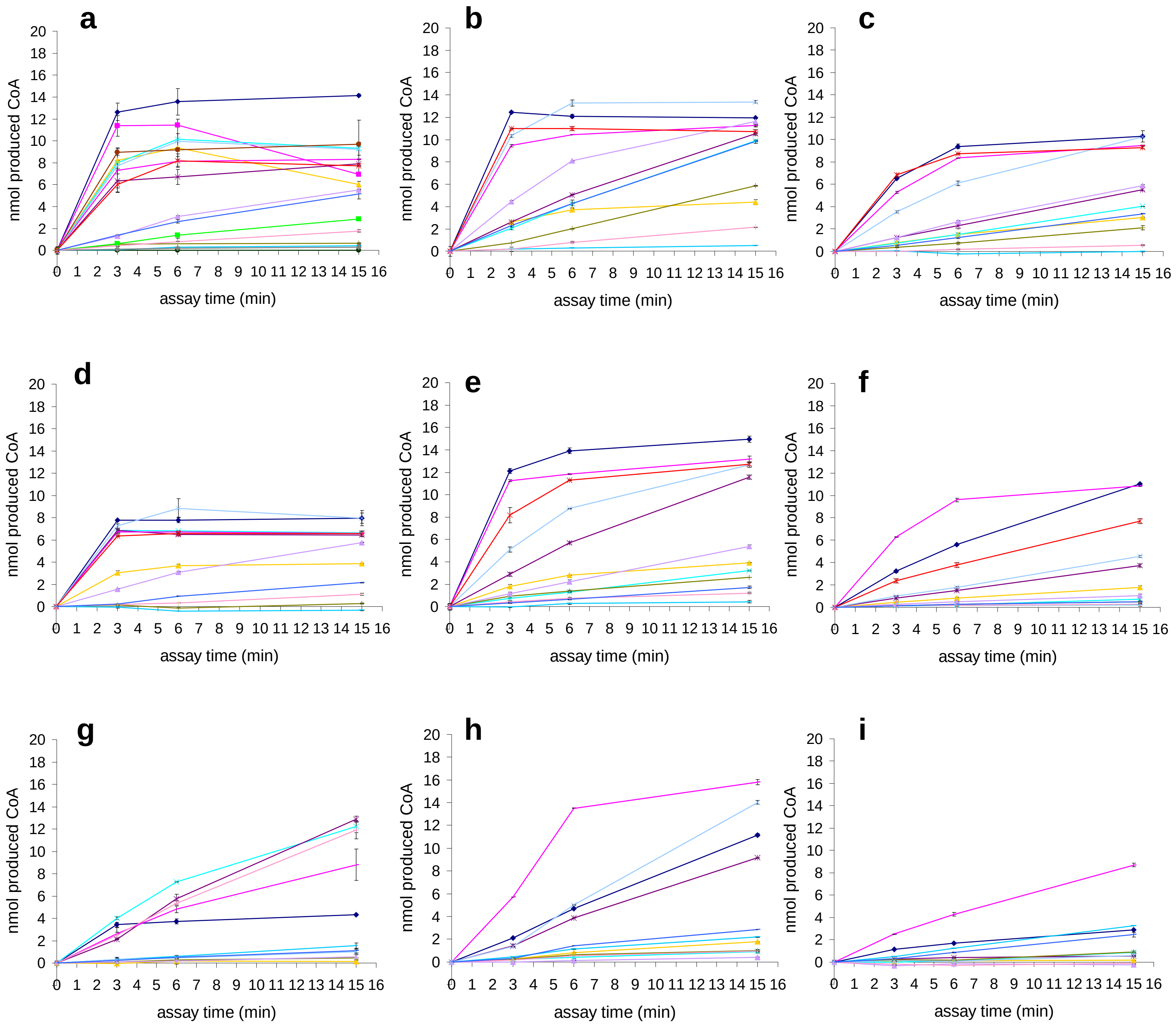
a
b
c
d
e
f
g
h
i
nmol produced CoA
assay time (min)
0
2
4
6
8
10
12
14
16
18
20
0 1 2 3 4 5 6 7 8 9 10 11 12 13 14 15 16

FIG. S5, contd.

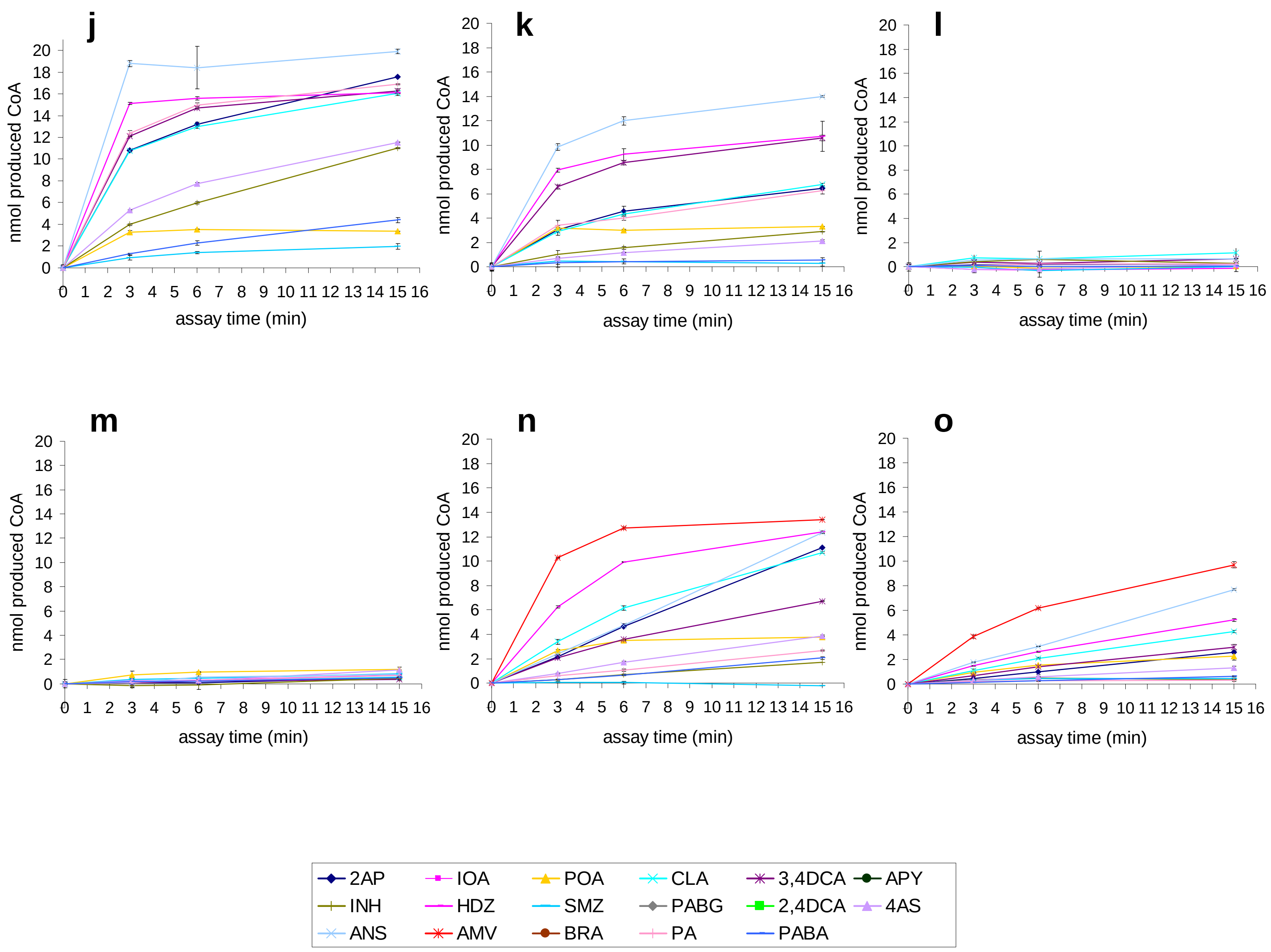
j
k
l
m
n
o
nmol produced CoA
assay time (min)
2AP
IOA
POA
CLA
3,4DCA
APY
INH
HDZ
SMZ
PABG
2,4DCA
4AS
ANS
AMV
BRA
PA
PABA

Supplementary Table 1: Oligos used for amplification and cloning of fungal *NAT* sequences.

Target[1]	Oligo Name[2]	Sequence (5'→3')[3]	Orientation	Application
(GIBM7)*NAT1*	FVEG_NAT1F	ATGGCGCGTCTCGAGGAT	Forward	Exon-intron annotation; ORF cloning
	FVEG_NAT1R	CTAGGCAAGTCTCTTCTCTTGAGGC	Reverse	Exon-intron annotation; ORF cloning
(GIBM7)*NAT2*	FVEG_NAT2F	ATGTCAGCCATATATTCTGAGGC	Forward	Exon-intron annotation; ORF cloning
	FVEG_NAT2R	CTACTCGCTCTTAATCTCAGTCTGG	Reverse	Exon-intron annotation; ORF cloning
(GIBM7)*NAT3*	FVEG_NAT3F	CAACATGGCGGATCGAAT	Forward	Exon-intron annotation
	FVEG_NAT3F(full_orf)	ATGGCGGATCGAATTCGATA	Forward	ORF cloning (pGEM vector)
	FVEG_NAT3R	TATGATCCAGTTCTCCGGTATGA	Reverse	Exon-intron annotation
	FVEG_NAT3R(full_orf)	CTATGATCCAGTTCTCCGGTAT	Reverse	ORF cloning (pGEM vector)
	VERTI3-MT-NDEI-F	GGCCGCGGGAATTCGCATATGGCGGATCGAATTCG	Forward	ORF cloning (pET28 vector)
	VERTI3-MT-NOTI-R	CGACCTGCAGGCGGCCGCGAATTCACTAGTGATTCTATGATCCAG	Reverse	ORF cloning (pET28 vector)
(GIBM7)*NAT4*	FVEG_NAT4F	ATGGCTTCCGCATATAGTCAAG	Forward	Exon-intron annotation; Cloning
	FVEG_NAT4R_BROAD	TTATGGTACAGTGCCTCTGAGC	Reverse	Exon-intron annotation; Cloning
	FVEG_NAT4R_FGENESH	TCAGGCAAAGTCATCACCATC	Reverse	Exon-intron annotation
(GIBZE)*NAT1*	FGSG_NAT1F	ATGTCGTGTCTTCCCGACC	Forward	Exon-intron annotation; ORF cloning
	FGSG_NAT1R	TCAAGCTAGTCTTCTCTCCTCTGG	Reverse	Exon-intron annotation; ORF cloning
(GIBZE)*NAT2*	FGSG_NAT2F	ATGTCGGCTATATACTCAGAAGCC	Forward	Exon-intron annotation; ORF cloning
	FGSG_NAT2R	TTACTCGCTCTTAATCTCAGTTTGG	Reverse	Exon-intron annotation; ORF cloning
(GIBZE)*NAT3*	FGSG_NAT3F	ATGATGGAAAGAATCAAGTACAGTGAG	Forward	Exon-intron annotation; ORF cloning
	FGSG_NAT3R	CTACGGTATAGCACCAGCCG	Reverse	Exon-intron annotation; ORF cloning

(FUSO4)*NAT1*	FOXG_NAT1F	ATGGCGCGTCTCGAAGAT	Forward	Exon-intron annotation; ORF cloning
	FOXG_NAT1R	TTATACCTTGCTCTGCCCAAGT	Reverse	Exon-intron annotation; ORF cloning
(FUSO4)*NAT2*	FOXG_NAT2F	ATGTCAGCCATTTATTCTGAAGCC	Forward	Exon-intron annotation; ORF cloning
	FOXG_NAT2R	CTACTGGGTCTTAATCTCAGTCTGG	Reverse	Exon-intron annotation; ORF cloning
	OXY2-INS-F	CGGGAATTCGATTA**TG**TCAGCCATTTATTC	Forward	Correction of PCR introduced mutation to clone
	OXY2-INS-R	GAATAAATGGCTG**AC**ATAATCGAATTCCCG	Reverse	Correction of PCR introduced mutation to clone
(FUSO4)*NAT3*	FOXG_NAT3F	ATGTCTGGTACTCGTATAAACTTCTCT	Forward	Exon-intron annotation; ORF cloning
	FOXG_NAT3R	CTATGGTATAGCGCCCGCA	Reverse	Exon-intron annotation; ORF cloning
(FUSO4)*NAT4*	FOXG_NAT4F	ATGACTTCAGCATATAGTCAAGAGC	Forward	Exon-intron annotation; ORF cloning
	FOXG_NAT4R	TTATGGCATAGTGCCTCTGAGC	Reverse	Exon-intron annotation; ORF cloning
(ASPFN)*NAT1*	AFL_NAT1F_BROAD	TTAATCAGTACGGGGCGAAC	Forward	Exon-intron annotation
	AFL_NAT1F_FGENESH	ATGTGCGGTGTCAAAGGTTAG	Forward	Exon-intron annotation
	AFL_NAT1F(full_orf_ss)	ATGTGCGGTGTCAAAGACG	Forward	Cloning
	AFL_NAT1R_ORF	CCTCTTTTCTCCTCCTCTGTCA	Reverse	Exon-intron annotation
	AFL_NAT1R_PARTIAL	TGGGAGCTTGATACACTCGAA	Reverse	Exon-intron annotation
	AFL_NAT1R(full_orf_ss)	TTACGCTAACATCTGATCCTCTGA	Reverse	Cloning
(ASPFN)*NAT2*	AFL_NAT2F	ATGGCTTCCCCATCCCA	Forward	Exon-intron annotation; ORF cloning
	AFL_NAT2R	CTATTTAATCTGAGATACTAAGCCCCG	Reverse	Exon-intron annotation; ORF cloning
	FLA2-MT-F	GCGTGGGGAG**A**CAGAGACGGTG	Forward	Correction of PCR introduced mutation to clone
	FLA2-MT-R	CACCGTCTCTG**T**CTCCCCACGC	Reverse	Correction of PCR introduced mutation to clone
(ASPFN)*NAT3*	AFL_NAT3F	GCCTACTCAGCGCTTCAGAT	Forward	Exon-intron annotation

	AFL_NAT3F(full_orf)	ATGAGCTCCGCCTACTCAG	Forward	ORF cloning (pGEM vector)
	AFL_NAT3R	CAAATCGAGAACCATTTCCG	Reverse	Exon-intron annotation
	AFL_NAT3R(full_orf)	TCAATCAAGACAAATCGAGAACCA	Reverse	ORF cloning (pGEM vector)
	FLA3-NDEI-F	GGCCGCGGGCATATGAGCTCCG	Forward	ORF cloning (pET28 vector)
	FLA3-MT-NOTI-R	CGACCTGCAGGCGGCCGCACTAGTGATTTCAATCAAGACAAATC	Reverse	ORF cloning (pET28 vector)
(ASPFN)*NAT4*	AFL_NAT4F_FGENESH	ATGTCCTTCTGTTTCTGGGATTT	Forward	Exon-intron annotation; Cloning
	AFL_NAT4F_GENEID	GGTTGGCGTACGAACCTCT	Forward	Exon-intron annotation
	AFL_NAT4R	TTAACTATCGCTCGTCGCC	Reverse	Exon-intron annotation; Cloning
(EMENI)*NAT1*	AN_NAT1F	ATGGCGTCAACATTCACTCG	Forward	Exon-intron annotation; ORF cloning
	AN_NAT1R	CTACTTCAATTCAGACACTAGACCCT	Reverse	Exon-intron annotation; ORF cloning
pGEM & pET28 vectors	T7-promoter	TAATACGACTCACTATAGGG	Forward	Sequencing of clones
pET28 vector	T7-terminator	GCTAGTTATTGCTCAGCGG	Reverse	Sequencing of clones
pGEM vector	SP6	ATTTAGGTGACACTATAGAA	Reverse	Sequencing of clones
Adaptor oligo[4]	SacII_NdeI_adF	TATGAGTAGCGGC	Forward	*Nde*I-*Sac*II ligation adaptor
Adaptor oligo[4]	SacII_NdeI_adR	CGCTACTCA (phosphorylated at the 5'-end)	Reverse	*Nde*I-*Sac*II ligation adaptor

[1] UniProt Taxonomy organism-specific mnemonics GIBM7, GIBZE, FUSO4, ASPFN and EMENI are used as prefixes to the symbols of *NAT* genes from sequenced strains of *F. verticillioides* (*G. moniliformis*), *F. graminearum* (*G. zeae*), *F. oxysporum* f.sp. *lycopersici*, *A. flavus* and *A. nidulans* (*E. nidulans*), respectively. Details of vector-specific primers and of adaptor oligos used for ligation into the pET28b(+) vector are also provided.

[2] Oligos were provided by VBC Biotech (Austria) or Sigma-Genosys (U.K.).

[3] Initiation and stop codons (predicted as described in Glenn *et al.* (2010), *FEBS Lett.* 584: 3158-3164) are shaded gray in oligos used for amplification of full-length *NAT* ORFs. Where present, the recognition sequences of *Nde*I (CATATG) and *Not*I (GCGGCCGC) restriction endonucleases are underlined. Primers designed to correct PCR-introduced mutations in two clones are shown with the modified nucleotide(s) in bold (site-directed mutagenesis was carried out with the QuikChange II kit by Agilent Technologies, according to the manufacturer's instructions).

[4] The two complementary adaptor oligos were mixed in equimolar amounts, at concentration of 0.5-1 μg/μl (1-10 OD_{260} units) in 10 mM Tris-HCl (pH 8.0), 50 mM NaCl and 1 mM EDTA. The mixture was heated to 94 ^{o}C and slowly (1 ^{o}C/min) cooled to room temperature. The annealed oligos were added to each ligation reaction at 20- to 50-molar excess relative to the compatible DNA fragment (insert), and ligation took place at 4 ^{o}C overnight. The incorporated adaptor introduced 4 extra amino acid residues (Met-Ser-Ser-Gly) between the *N*-terminal hexa-histidine tag and the NAT proteins expressed from the pET28b(+) vector.
